# TRIM25‐Mediated INSIG1 Ubiquitination Promotes MASH Progression Through Reprogramming Lipid Metabolism

**DOI:** 10.1002/advs.202414646

**Published:** 2025-04-15

**Authors:** Hao Zhang, Xiangxu Kong, Wei Wang, Huaxin Zhou, Haoran Qu, Zhengyao Guan, Honglei Wu, Xiangyu Zhai, Bin Jin

**Affiliations:** ^1^ Organ Transplant Department Qilu Hospital of Shandong University Jinan China; ^2^ Shandong Province Engineering Research Center for Multidisciplinary Research on Hepatobiliary and Pancreatic Malignant Tumors Jinan China; ^3^ Department of Hepatobiliary Surgery the Second Hospital of Shandong University Beiyuan Street & 247 Jinan Shandong China; ^4^ Medical Integration and Practice Center Shandong University Jinan China; ^5^ Department of Gastroenterology the Second Hospital of Shandong University Beiyuan Street & 247 Jinan Shandong China

**Keywords:** C_27_H_26_N_2_O_4_S, exosomes, lipid metabolism, MASH, targeted delivery, TRIM25, ubiquitination

## Abstract

The global incidence of Metabolic dysfunction‐associated steatohepatitis (MASH) is increasing, highlighting the urgent need for new treatment strategies. This study aimed to investigate the involvement of tripartite motif‐containing 25 (TRIM25) in MASH progression and explore the therapeutic potential of the TRIM25 inhibitor, C_27_H_26_N_2_O_4_S. Functional studies reveal that TRIM25 promoted lipid accumulation and inflammation by ubiquitinating and degrading insulin‐induced gene 1 (INSIG1), thereby enhancing the nuclear translocation of sterol regulatory element‐binding protein 2 (SREBP2) and upregulating lipid biosynthesis genes. In vivo experiments using TRIM25 knockout mice demonstrated that TRIM25 deletion ameliorated MASH progression, reduced fibrosis, and decreased inflammatory cell infiltration. It identifies C_27_H_26_N_2_O_4_S as a specific inhibitor of TRIM25. C_27_H_26_N_2_O_4_S effectively decreased INSIG1 ubiquitination and attenuated lipid accumulation in the hepatocytes. To enhance the hepatic delivery of C_27_H_26_N_2_O_4_S, it utilizes exosomes derived from hepatic stellate cells (HSC‐EVs). Biodistribution analysis confirmed that the HSC‐EVs preferentially accumulated in the liver. In a MASH mouse model, HSC‐EV‐encapsulated C_27_H_26_N_2_O_4_S (C_27_H_26_N_2_O_4_S@HSC‐EV) significantly reduced hepatic lipid accumulation and alleviated MASH severity and fibrosis. This study highlights the critical regulatory role of TRIM25 in MASH and presents C_27_H_26_N_2_O_4_S@HSC‐EV as a promising therapeutic approach for MASH treatment.

## Graphical abstract

TRIM25‐mediated ubiquitination of INSIG1 serves as a key driver of lipid metabolism dysregulation in MASH progression. It identifies a novel small‐molecule inhibitor, C_27_H_26_N_2_O_4_S, which effectively inhibits TRIM25 activity, reducing lipid accumulation and inflammation. The exosome‐mediated delivery of C_27_H_26_N_2_O_4_S to the liver enhances targeting and therapeutic potential, offering innovative insights into MASH treatment.

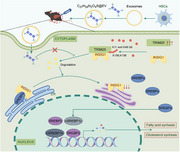

## 1 Introduction

Metabolic dysfunction‐associated steatohepatitis (MASH) is a crucial phase within metabolic dysfunction‐associated steatotic liver disease (MASLD).^[^ ^1^, ^2^ ^]^ It is distinguished by inflammation, hepatic steatosis, and different levels of fibrosis, which can advance to cirrhosis and hepatocellular carcinoma.^[^ ^3^, ^4^, ^5^ ^]^ The prevalence of MASH is rising globally, paralleling the obesity epidemic, which highlights the pressing necessity for an enhanced understanding of its pathogenesis and formulation of effective therapeutic strategies.^[^ ^6^, ^7^ ^]^ Recent advancements in MASH research have elucidated the pathophysiological mechanisms underlying its progression, highlighting the critical roles of lipid metabolism, inflammation, and cellular signaling pathways.^[^ ^8^, ^9^, ^10^ ^]^ Dysregulated lipid metabolism leads to the excessive accumulation of lipids in hepatocytes, inducing lipotoxicity, oxidative stress, and inflammatory responses. Cholesterol homeostasis, governed by intricate regulatory networks, is also disrupted in MASH, exacerbating liver injury and fibrosis.^[^ ^11^, ^12^ ^]^ Despite these advances, the molecular mechanisms underlying MASH progression are not fully understood and require further investigation.

Ubiquitination, a post‐translational modification process, is essential for controlling protein stability and function, thereby influencing numerous cellular pathways.^[^ ^13^, ^14^ ^]^ The involvement of ubiquitination in MASH progression is an emerging area of research focusing on the roles of specific ubiquitin ligases.^[^ ^15^, ^16^ ^]^ Tripartite motif‐containing 25 (TRIM25) is an E3 ubiquitin ligase that facilitates ubiquitination and the subsequent proteasomal degradation of target proteins. TRIM25 has been implicated in various cellular processes, including antiviral responses, cell proliferation, and apoptosis.^[^ ^17^, ^18^, ^19^, ^20^ ^]^ However, its role in metabolic regulation and liver diseases, particularly MASH, is an emerging area of interest.

Lipid and cholesterol metabolism play pivotal roles in MASH pathogenesis. Dysregulating these metabolic pathways leads to excessive lipid accumulation, inflammatory responses, and oxidative stress within the liver, driving the progression from simple steatosis to MASH.^[^ ^21^, ^22^, ^23^ ^]^ Sterol regulatory element‐binding proteins (SREBPs) are transcription factors that control the expression of genes involved in cholesterol and fatty acid synthesis.^[^ ^24^, ^25^ ^]^ Insulin‐induced gene 1 (INSIG1) inhibits SREBP2 activation by retaining it in the endoplasmic reticulum.^[^ ^26^, ^27^ ^]^ Understanding the molecular interplay among ubiquitination, lipid metabolism, and MASH progression is crucial for identifying potential therapeutic targets. In this study, the significance of TRIM25 in MASH is underscored by its ability to ubiquitinate INSIG1. The ubiquitination and degradation of INSIG1 by TRIM25 facilitates the activation and nuclear translocation of SREBP2. Upon entering the nucleus, SREBP2 facilitates the transcription of genes associated with cholesterol and fatty acid biosynthesis, thereby contributing to the lipid accumulation and metabolic disturbances characteristic of MASH.

In our study, we identified a small‐molecule inhibitor, C_27_H_26_N_2_O_4_S, through virtual screening, which specifically inhibits the ubiquitin ligase activity of TRIM25. By inhibiting TRIM25, C_27_H_26_N_2_O_4_S prevents INSIG1 degradation, thus reducing the activation of SREBP2 and subsequent lipid accumulation in hepatocytes. This inhibition offers a potential therapeutic approach to mitigate MASH progression by targeting a critical regulatory axis in lipid metabolism.

This study aimed to elucidate the role of TRIM25‐mediated ubiquitination of INSIG1 in regulating SREBP2 activity and its impact on hepatic lipid metabolism and MASH progression. Identifying and characterizing C_27_H_26_N_2_O_4_S as a TRIM25 inhibitor underscores the therapeutic potential of modulating ubiquitination pathways for MASH treatment. This study aimed to offer a novel understanding of the molecular processes by which TRIM25 controls lipid metabolism. Additionally, we investigated the potential of TRIM25 inhibition as an innovative therapeutic strategy for MASH.

## 2 Results

### 2.1 TRIM25 was Upregulated in MASH and Correlated with MASH Progression

**Figure 1:**
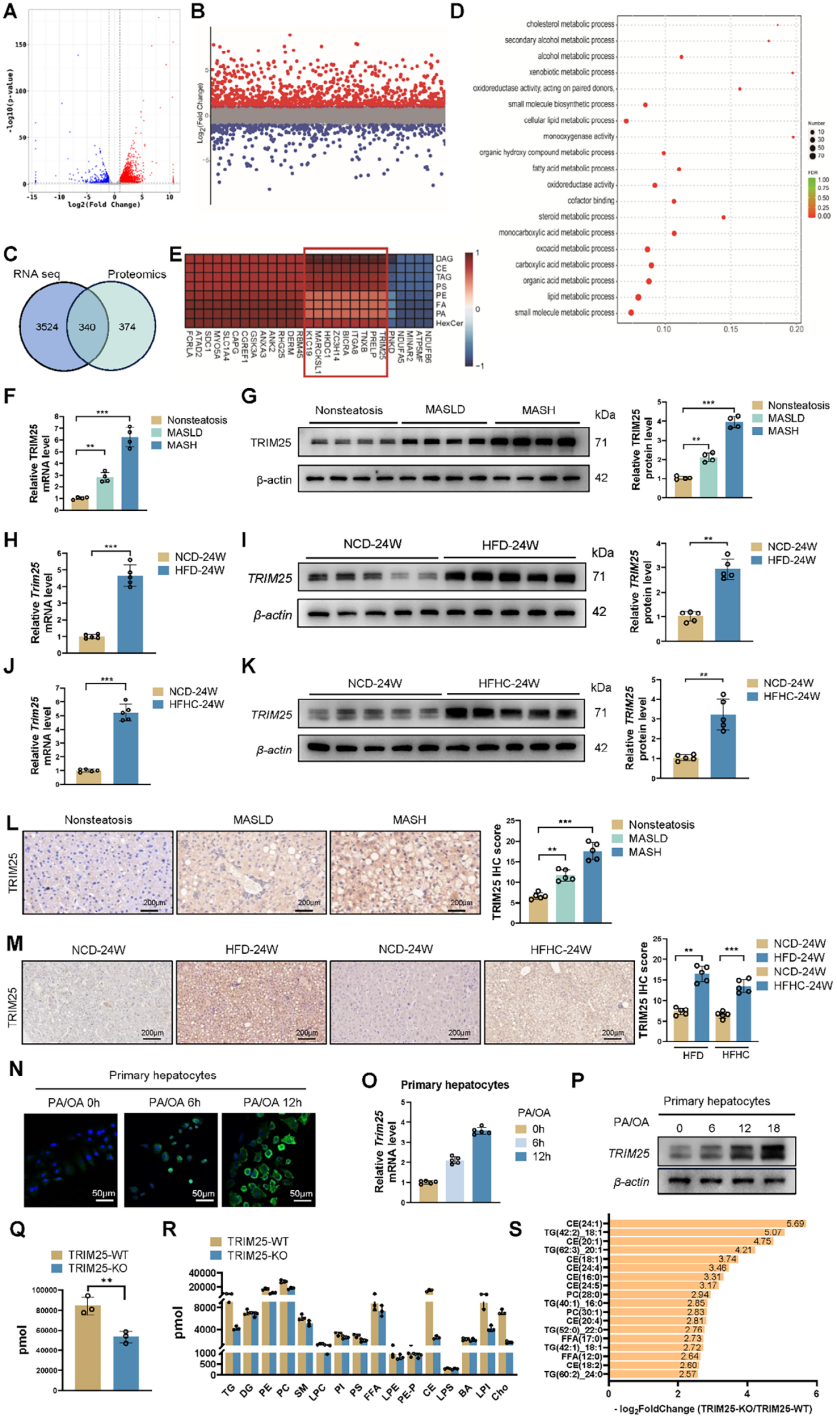
TRIM25 upregulates in hepatocytes during MASH pathogenesis. A) Transcriptomic analysis of liver samples from MASH and normal tissues identified 3864 differentially expressed genes. B) Proteomic analysis identified 714 differentially expressed proteins between MASH and normal liver samples (*p* < 0.05, log_2_FC > 1). C) Venn diagram revealing 340 significantly differentially expressed genes in the transcriptomic and proteomic analyses. D) Functional enrichment analysis of the 340 genes. E) Integrated proteomic and metabolomic analyses identified genes significantly correlated with cholesterol and triglycerides. F and G) TRIM25 expression was measured in human normal liver tissue, MAFLD, and MASH liver tissue using RT‐qPCR (F) and WB analysis G). H,I) TRIM25 expression was assessed using RT‐qPCR (H) and WB (I) analysis in the liver tissues of mice from the HFD‐induced MASH mouse model and control mouse (5 vs 5 mice). J,K) TRIM25 expression was assessed in the liver tissues of mice from the HFHC‐induced MASH mouse model and control mouse (5 vs 5 mice). L) Immunohistochemical analysis confirmed increased TRIM25 expression in human MASH livers. M) Immunohistochemical analysis in HFD or HFHC‐induced MASH mouse. N) TRIM25 mRNA expression in a lipid toxicity model induced in cells with PA/OA. O) TRIM25 protein levels in cell lipid toxicity model. P) Immunofluorescence staining was performed to visualize the expression of TRIM25. Q) Lipidomic analysis of TRIM25‐WT and TRIM25‐KO MIHA cells revealed a significant reduction in total lipid content in TRIM25‐KO cells (3 vs 3). R) Differences in the levels of various lipid classes. S) The log_2_FoldChange of the most significantly altered lipid species. Data are presented as mean ± SD. *p < 0.05, **p < 0.01, and ***p < 0.001.

To elucidate the critical molecules involved in the onset and progression of MASH, we obtained three pathologically confirmed MASH tissue samples and three normal liver tissue samples from the Second Hospital of Shandong University. Comprehensive multi‐omics analyses were performed on six liver samples. Transcriptomic analysis identified 3864 differentially expressed genes (**Figure** **1**A), while proteomic analysis identified 714 differentially expressed proteins (Figure 1B; *p* < 0.05, log_2_FoldChange>1). Based on these results, we identified 340 differentially expressed genes at both the mRNA and protein levels (Figure 1C). Functional enrichment analysis of these 340 genes revealed significant enrichment in pathways related to cholesterol and other lipid metabolism (Figure 1D). Subsequently, integrated proteomic and metabolomic analyses identified genes significantly correlated with cholesterol and triglycerides, including keratin 19 (K1C19), MARCKS like 1 (MARCKSL1), hexokinase domain containing 1 (HKDC1), and other genes (Figure 1E). Correlation analysis of these genes with those enriched in metabolic pathways demonstrated that TRIM25 was significantly correlated with metabolic genes in the transcriptomic and proteomic data (Figure S1A,B, Supporting Information). To further confirm the association between TRIM25 and MASH progression, liver tissues were collected from patients with MASLD and MASH. The mRNA and protein levels of TRIM25 were significantly higher in MASLD livers compared to healthy livers, and TRIM25 expression was markedly higher in MASH livers compared to MASLD livers. This indicates a progressive increase in TRIM25 expression from normal to MASLD to MASH, correlating positively with the severity of MASH (Figure 1F,G). Moreover, we induced MASH in mice using a high‐fat diet (HFD) or a high‐fat and high‐cholesterol (HFHC) diet and measured TRIM25 expression levels (Figure S2A, Supporting Information). TRIM25 expression was significantly elevated in the livers of HFD‐induced MASH mice compared to control mice fed a normal chow diet (NCD) (Figure 1H,I). Similarly, in the HFHC‐induced MASH mouse model, we detected an upregulation of TRIM25 expression (Figure 1J,K). Immunohistochemical analysis further confirmed the increase in TRIM25 expression in MASH livers, consistent with the polymerase chain reaction (PCR) and western blot (WB) results (Figure 1L and 1). Furthermore, a lipid toxicity model was induced in cells using palmitic acid (PA) and oleic acid (OA) (Figure 1N). TRIM25 mRNA and protein expression levels were positively correlated with the duration of PA/OA induction (Figure 1O,P). We performed lipidomic analyses on TRIM25‐knockout (TRIM25‐KO) and wild‐type (TRIM25‐WT) MIHA cells to further determine the regulatory role of TRIM25 in lipid metabolism. The results revealed that the total lipid content was significantly reduced in TRIM25‐KO cells (Figure 1Q). Among the lipid species, triglycerides (TG), cholesterol esters (CE), and cholesterol (Cho) exhibited the most pronounced changes (Figure 1R). Notably, among the 18 most significantly altered lipid species, the majority belonged to TG, CE, free fatty acids (FFA), and Cho (Figure 1S). These findings demonstrate that TRIM25 expression progressively increases during the transition from normal liver to MASLD to MASH, highlighting its potential role in disease progression.

### 2.2 TRIM25 Promoted Lipid Accumulation and Inflammation in Hepatocytes

**Figure 2:**
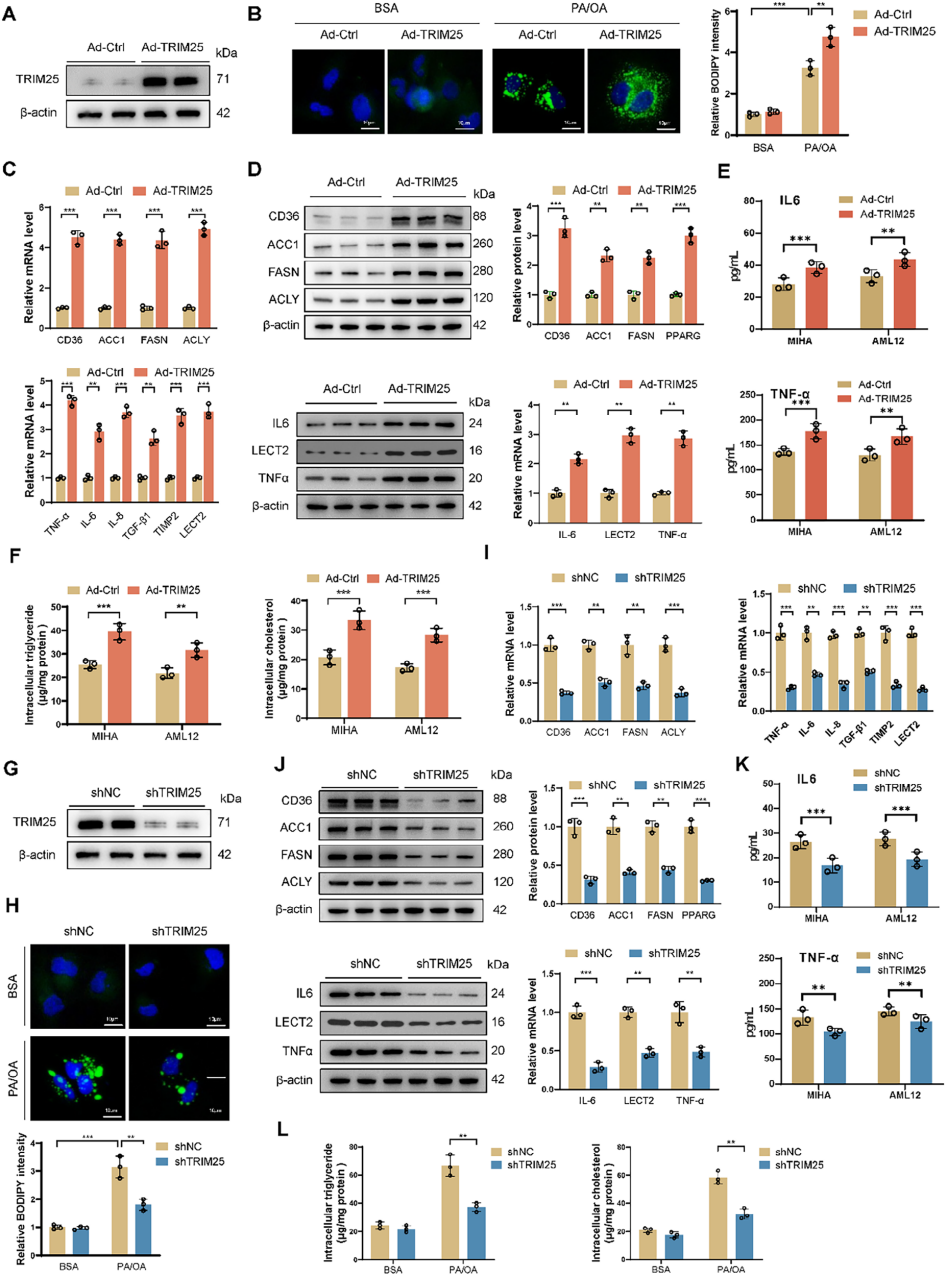
TRIM25 promotes lipid accumulation and inflammation in hepatocytes. A) TRIM25‐overexpressing hepatocyte cell lines generated using adenovirus. B) BODIPY staining revealed increased lipid content in TRIM25‐overexpressing MIHA hepatocytes. C,D) TRIM25 overexpression significantly upregulated mRNA (C) and protein (D) levels of lipid metabolism‐related genes and proinflammatory genes in MIHA. E) Overexpression of TRIM25 significantly increased the levels of IL‐6 and TNF‐*α* in the cell culture medium, as determined by ELISA assays. This observation suggests that TRIM25 may play a key role in promoting the secretion of these pro‐inflammatory cytokines, potentially implicating its involvement in the regulation of inflammatory responses. F) The assay kits detected that cholesterol and triglyceride levels were significantly elevated in TRIM25‐overexpressing cells. G) WB was performed to validate the knockdown efficiency of TRIM25 in hepatocytes. H) BODIPY fluorescence staining was used to detect intracellular lipid changes following TRIM25 knockdown in MIHA cells. I,J) TRIM25 knockdown significantly downregulated mRNA (I) and protein (J) levels of lipid metabolism‐related and proinflammatory genes in MIHA cells. K) Knockdown of TRIM25 significantly increased the levels of IL‐6 and TNF‐*α* in the cell culture medium, as determined by ELISA assays. L) Reduced intracellular cholesterol and triglyceride levels in TRIM25 knockdown MIHA cells. Data are presented as mean ± SD. *p < 0.05, **p < 0.01, and ***p < 0.001.

To determine the role of TRIM25 in lipid metabolism in MASH, we generated TRIM25‐overexpressing hepatocyte cell lines using adenovirus and established a cellular lipid toxicity model (**Figure** **2**A,B). BODIPY staining revealed that TRIM25 overexpression significantly increased hepatocyte lipid content (Figure 2B). We assessed the expression levels of lipid metabolism and inflammation‐related genes. We found that TRIM25 significantly upregulated the mRNA and protein levels of lipid metabolism‐related genes (CD36, ACC1, FASN, and PPARG) and proinflammatory genes (interleukin‐6, LECT2, and tumor necrosis factor‐*α*) (Figure 2C,D). Overexpression of TRIM25 significantly increased the levels of IL‐6 and TNF‐*α* in the cell culture medium, as determined by ELISA assays (Figure 2E). Additionally, cholesterol and triglyceride levels were significantly elevated in TRIM25‐overexpressing cells (Figure 2F).

Moreover, the expression of genes related to fatty acid synthesis and genes involved in cholesterol synthesis and uptake was significantly increased in cells overexpressing TRIM25 (Figure S2C, Supporting Information). Conversely, in hepatocytes with stable TRIM25 knockdown, we observed a significant reduction in lipid content (Figure 2G,H; Figure S2D, Supporting Information). TRIM25 knockdown also significantly downregulated the mRNA and protein levels of lipid metabolism‐related and proinflammatory genes (Figure 2I,J), increased the levels of IL‐6 and TNF‐*α* in the cell culture medium (Figure 2K), and reduced intracellular cholesterol and triglyceride levels, along with the expression of related genes (Figure 2L; Figure S2E, Supporting Information). These results suggest that TRIM25 facilitates lipid build‐up and inflammation in liver cells, emphasizing its possible involvement in MASH development.

### 2.3 TRIM25 Knockout Attenuated MASH Progression, Lipid Accumulation, and Inflammatory Response In Vivo

**Figure 3:**
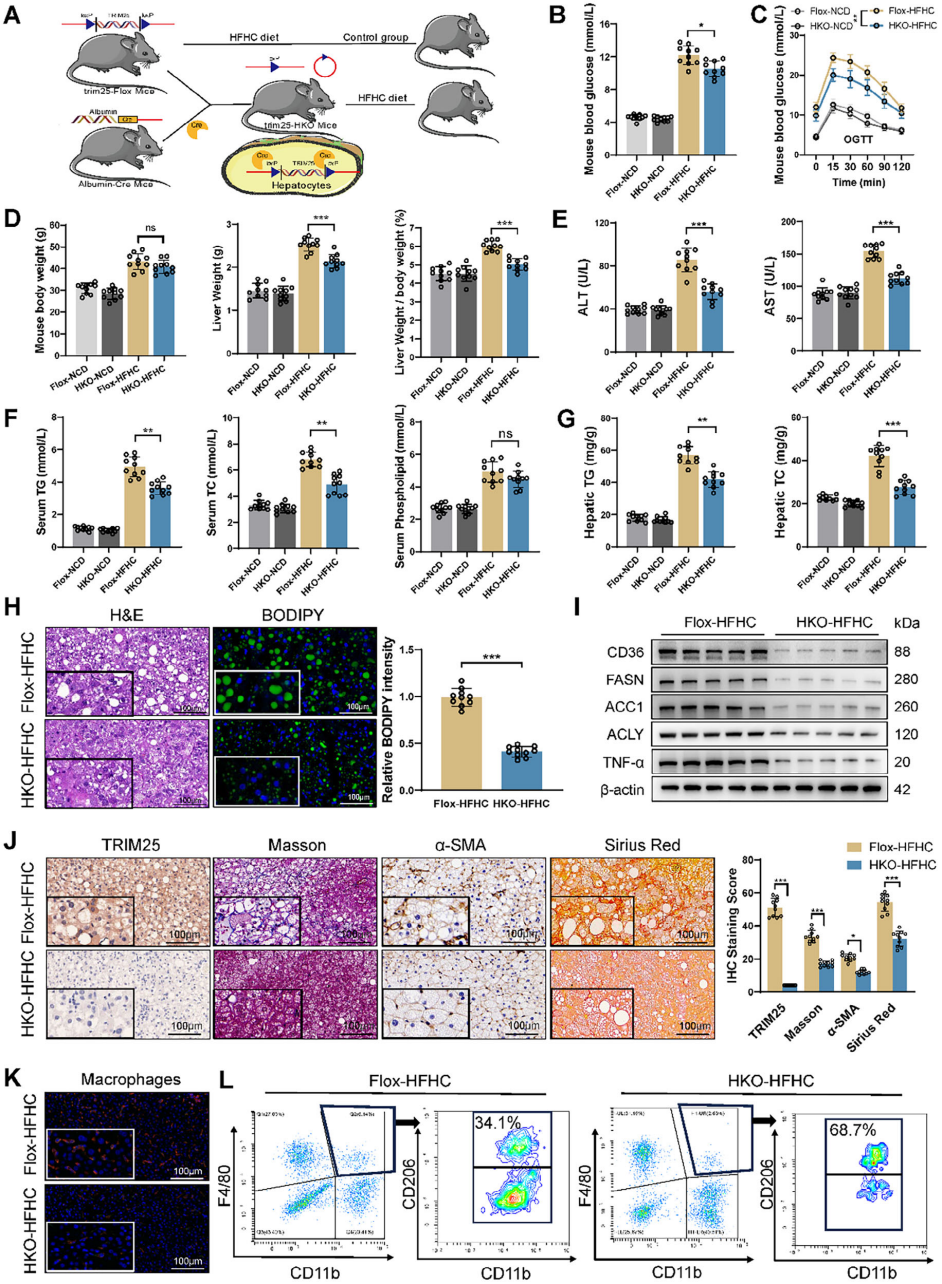
TRIM25 deletion attenuates MASH progression and inflammation in vivo. A) Generation of hepatocyte‐specific Trim25 knockout (HKO) mice. B–D) HFHC‐induced HKO‐HFHC MASH model mice exhibited lower blood glucose levels (B), improved glucose tolerance (C), lower liver weight, and liver weight/body weight ratio (D) compared to controls (Flox‐HFHC) (n = 10 each group). E) Serum ALT and AST levels were significantly reduced in HKO‐HFHC mice. F,G) Serum and liver cholesterol and triglyceride levels were markedly lower in HKO‐HFHC mice. H) BODIPY staining confirmed reduced hepatic lipid accumulation in HKO mice. I) Trim25 knockout downregulated expression of lipid metabolism‐related genes. J) Masson's trichrome, *α*‐SMA, and Sirius Red staining indicated significantly lower liver fibrosis levels in HKO mice. K) Immunofluorescence analysis revealed reduced macrophage infiltration in HKO mice. L) Flow cytometry demonstrated the inhibition of M1 macrophage polarization and an increased proportion of anti‐inflammatory M2 macrophages in HKO mice. Data are presented as mean ± SD. *p < 0.05, **p < 0.01, and ***p < 0.001.

To explore the role of TRIM25 in MASH in vivo, we generated hepatocyte‐specific TRIM25 knockout (HKO) mice (**Figure** **3**A). MASH was induced in control (TRIM25‐Flox) and TRIM25‐HKO mice using an HFHC diet, followed by an assessment of various biochemical and histological parameters. HKO‐HFHC mice exhibited lower blood glucose levels, improved glucose tolerance, lower liver weight to body weight ratio compared to controls (Flox‐HFHC) (Figure 3B‐D). Serum ALT and AST levels were significantly reduced in HKO‐HFHC mice (Figure 3E). Serum and liver cholesterol and triglyceride levels were markedly lower in HKO‐HFHC mice, although a non‐significant difference was observed in inguinal fat weight (Figure 3F,G; Figure S4A, Supporting Information). Consistent results were observed in HFD‐induced mice, where TRIM25 knockout significantly ameliorated hepatic fat accumulation, liver injury, and glucose tolerance in MASH mice (Figure S4B‐F, Supporting Information). BODIPY staining confirmed that TRIM25 knockout reduced hepatic lipid accumulation and downregulated the expression of lipid metabolism‐related genes (Figure 3H,I; Figure S5A, Supporting Information). Fibrosis, a hallmark of MASH, was assessed using Masson's trichrome, *α*‐SMA, and Sirius Red staining. TRIM25 knockout mice exhibited significantly lower liver fibrosis levels compared to controls (Figure 3J). Macrophage infiltration and M1 polarization are critical for promoting MASH inflammation. Immunofluorescence analysis revealed that TRIM25 knockout significantly reduced macrophage infiltration (Figure 3K). Flow cytometry revealed that TRIM25 knockout inhibited M1 macrophage polarization and increased the proportion of anti‐inflammatory M2 macrophages (Figure 3L; Figure S4B, Supporting Information). Moreover, TRIM25 knockout reduced CD4+ T‐cell infiltration and activation (Figure S5C,D, Supporting Information). These findings suggest that TRIM25 deletion alleviates MASH progression and immune cell infiltration, highlighting its potential as a therapeutic target for MASH.

### 2.4 TRIM25 Directly Bound to INSIG1

**Figure 4:**
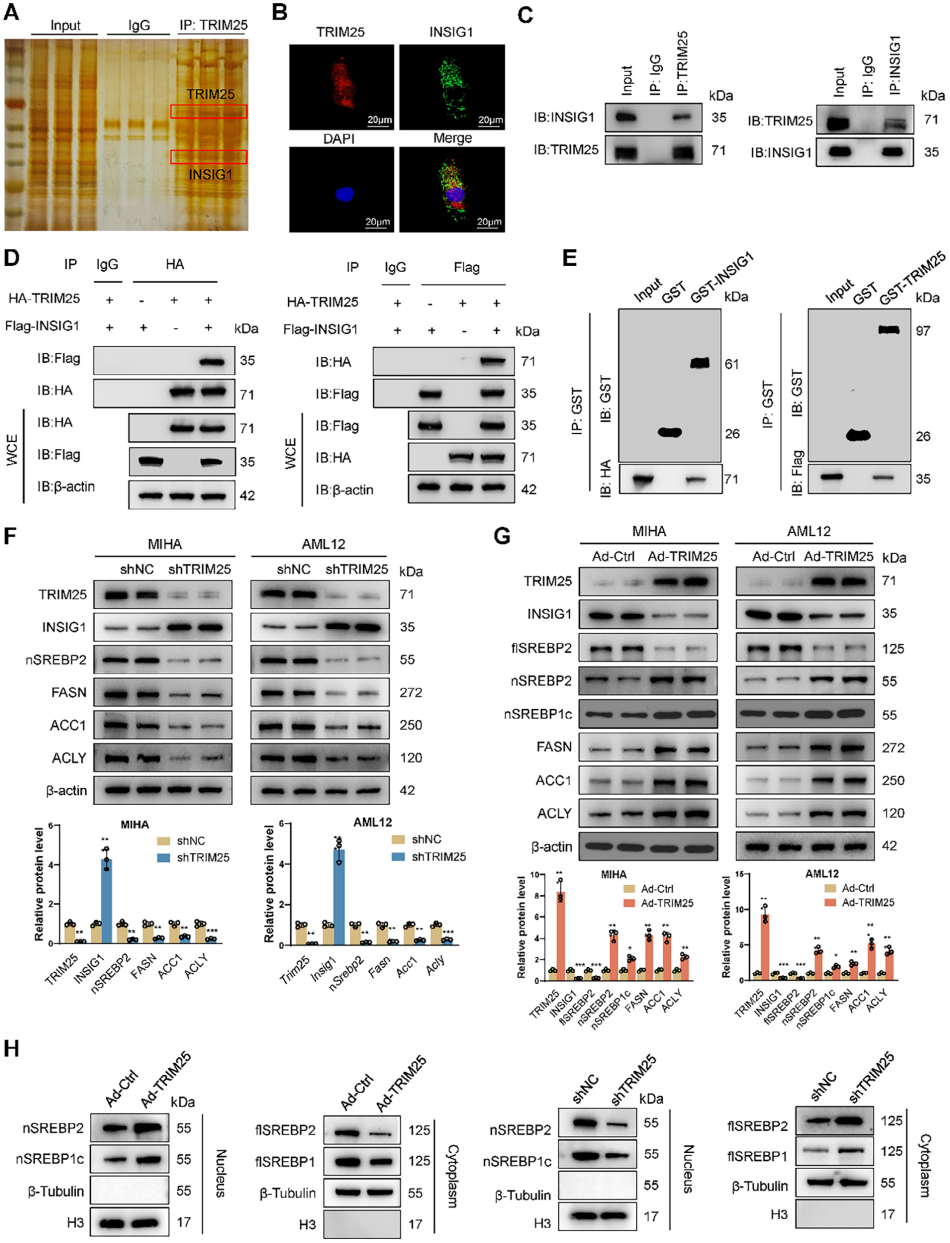
TRIM25 directly binds to INSIG1. A) Immunoprecipitation and silver staining. B) Immunofluorescence co‐localization revealed that TRIM25 and INSIG1 were predominantly distributed in the cytoplasm. C) Immunoprecipitation of MIHA cells confirmed the interaction between TRIM25 and INSIG1. D) HA‐TRIM25 and Flag‐INSIG1 were transfected into HEK293T cells, followed by co‐immunoprecipitation. E) GST pulldown assays demonstrated that GST‐fused TRIM25 pulled down INSIG1. F) TRIM25 knockdown upregulated INSIG1 protein levels and downregulated lipid metabolism‐related proteins and nuclear localization of SREBP2. G) TRIM25 overexpression decreased INSIG1 levels and upregulated lipid metabolism‐related proteins. H) After nucleocytoplasmic separation, the protein levels of SREBP2 in the cytoplasm and nucleus were measured. It was found that overexpression of TRIM25 promoted the nuclear translocation of SREBP2. Data are presented as mean ± SD. *p < 0.05, **p < 0.01, and ***p < 0.001.

To elucidate the molecular mechanism by which TRIM25 promotes MASH progression, we performed immunoprecipitation followed by mass spectrometry analysis of TRIM25 (**Figure** **4**A). Considering TRIM25's role as an E3 ubiquitin ligase that can ubiquitinate and degrade substrate proteins, we conducted immunoprecipitation and relative quantification of proteins in the control and TRIM25‐overexpressing cells. Of the 68 proteins identified to interact with TRIM25, 40 exhibited significant changes in the TRIM25‐overexpressing cells. INSIG1 exhibited the most significant decrease in TRIM25‐overexpressing cells (Table S3, Supporting Information), and previous studies have suggested that INSIG1 regulates the nuclear localization of the key lipid metabolism regulator SREBP2. Therefore, we hypothesized that TRIM25 interacts with INSIG1 to mediate its ubiquitination and subsequent degradation, thereby influencing hepatocyte lipid metabolism. To confirm the interaction between TRIM25 and INSIG1, we first performed immunofluorescence co‐localization, which revealed that both proteins were predominantly distributed in the cytoplasm (Figure 4B). Immunoprecipitation of MIHA cell lines further confirmed this interaction (Figure 4C). In HEK293T cells, co‐transfection with HA‐TRIM25 and Flag‐INSIG1 plasmids, followed by immunoprecipitation, confirmed this interaction (Figure 4D). To determine whether the interaction between TRIM25 and INSIG1 is direct, we conducted in vitro pulldown assays using proteins purified from *Escherichia coli*. GST pulldown assays demonstrated that GST‐fused TRIM25, but not GST alone, could pull down Flag‐INSIG1. Similarly, GST‐fused INSIG1 pulled down HA‐TRIM25 (Figure 4E). Having established that TRIM25 directly binds INSIG1, we investigated its regulatory effect of TRIM25 on INSIG1 protein expression. We observed that the TRIM25 knockdown resulted in the upregulation of INSIG1 and lipid metabolism‐related gene expression. Conversely, TRIM25 overexpression decreased the protein expression of these genes. Additionally, TRIM25 downregulated the full‐length SREBP2 (flSREBP2) protein while simultaneously upregulating the nuclear SREBP2 (nSREBP2) protein levels (Figure 4F,G). Further validation through nuclear‐cytoplasmic fractionation revealed that TRIM25 overexpression reduced the cytoplasmic protein content of flSREBPs and increased the protein levels of nSREBP1c and nSREBP2. Conversely, TRIM25 knockdown decreased the nuclear translocation of SREBP2 and nSREBP1c (Figure 4H).

### 2.5 TRIM25 Mediated Ubiquitin‐Dependent Degradation of INSIG1

**Figure 5:**
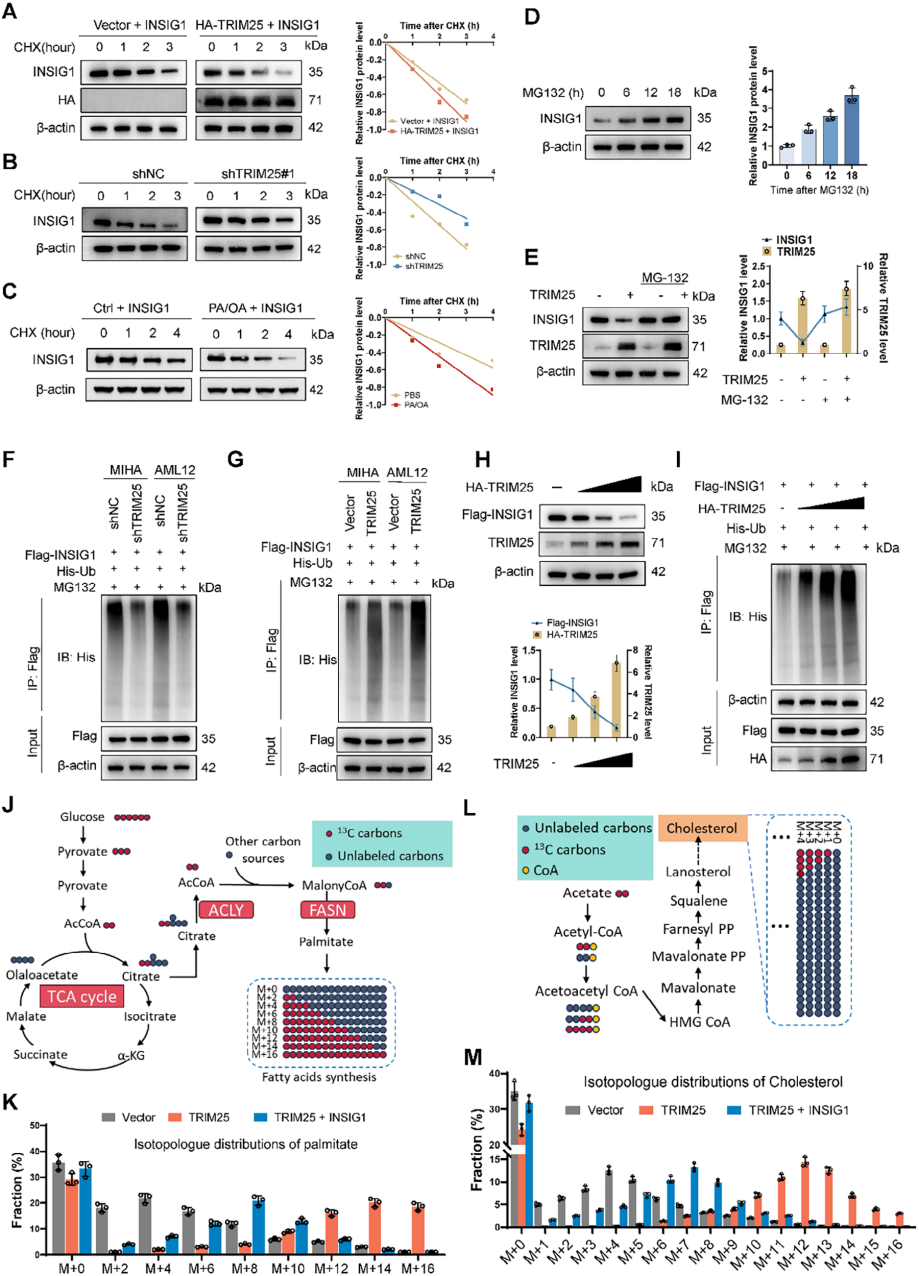
TRIM25 mediates ubiquitin‐dependent degradation of INSIG1. A) HA‐TRIM25 and INSIG1 plasmids were transfected into 293T cells, and the protein levels of INSIG1 were measured at 0, 1, 2, and 3 h after adding CHX. TRIM25 significantly promoted INSIG1 protein degradation. B) CHX was added to TRIM25 knockdown and control MIHA cells, and INSIG1 levels were measured at 0, 1, 2, and 3 h. C) In a cellular lipotoxicity model, INSIG1 degradation was accelerated. D) Treatment with the proteasome inhibitor MG132 increased INSIG1 protein levels, indicating degradation via the ubiquitin‐proteasome pathway. E) TRIM25 overexpression plasmid was transfected into MIHA cells, and the protein level of INSIG1 was detected with or without MG132. It was found that overexpression of TRIM25 did not reduce INSIG1 levels in the presence of MG132. F,G) Ubiquitination assays revealed that TRIM25 knockdown decreased INSIG1 ubiquitination (F), whereas TRIM25 overexpression enhanced it (G). H,I) INSIG1 degradation (H) and ubiquitination (I) levels exhibited a dose‐dependent relationship with TRIM25 expression in 293T cells. J) Schematic diagram shows the distribution of ^13^C‐labeled glucose through the tricarboxylic acid (TCA) cycle and de novo fatty acid synthesis metabolism. Control group, TRIM25 overexpression group, and TRIM25+INSIG1 overexpression MIHA cells were incubated with [U‐^13^C] glucose for 24 hours. K) The distribution of palmitic acid (C16:0) containing different numbers of ^13^C isotope carbons. L) Schematic representing the passage of ^13^C from ^13^C‐labeled acetate through the cholesterol biosynthetic pathway. Different treatment groups of MIHA cells were incubated with ^13^C‐labeled acetate for 48 hours. M) The distribution of palmitic acid (C16:0) containing different numbers of ^13^C isotope carbons. Data are presented as mean ± SD. *p < 0.05, **p < 0.01, and ***p < 0.001.

Previous results confirmed that TRIM25 directly binds to and negatively regulates INSIG1 protein levels. We examined protein stability and degradation rates to elucidate the molecular mechanism underlying this regulation. TRIM25 significantly promoted INSIG1 protein degradation. Conversely, upon TRIM25 knockdown, INSIG1 protein degradation was markedly reduced (**Figure** **5**A,B). In the cellular lipotoxicity model, we observed that INSIG1 degradation was accelerated (Figure 5C). Given that TRIM25 functions as an E3 ubiquitin ligase, we hypothesized that INSIG1 is regulated by ubiquitination. Treatment of cells with the proteasome inhibitor MG132 increased INSIG1 protein levels, indicating that INSIG1 was degraded via the ubiquitin‐proteasome pathway (Figure 5D). Furthermore, in the presence of MG132, overexpression of TRIM25 did not reduce INSIG1 levels, suggesting that TRIM25‐mediated INSIG1 degradation is ubiquitination‐dependent (Figure 5E). Ubiquitination assays revealed that TRIM25 knockdown decreased INSIG1 ubiquitination, whereas TRIM25 overexpression enhanced it (Figure 5F,G; Figure S5A,B, Supporting Information). Additionally, INSIG1 degradation and ubiquitination levels exhibited a dose‐dependent relationship with TRIM25 expression, characteristic of ubiquitin ligase activity (Figure 5H,I; Figure S5C, Supporting Information). We employed metabolic flux analysis to validate the role of TRIM25 and INSIG1 in the de novo synthesis of fatty acids and cholesterol. Cells were incubated with ^13^C‐labeled glucose, and the incorporation of the isotope into metabolic products was assessed (Figure 5J). Compared to control cells, TRIM25‐overexpressing cells exhibited a significant increase in the proportion of 13C‐labeled palmitic acid, with the isotopic peak appearing at M+14, indicating a substantial rightward shift relative to the control group's peak. Furthermore, overexpression of INSIG1 attenuated the TRIM25‐induced enhancement of fatty acid synthesis (Figure 5K). Similarly, cells were incubated with ^13^C‐labeled acetate to assess the distribution of ^13^C‐labeled cholesterol (Figure 5L). TRIM25 overexpression led to an increase in the synthesis of 13C‐labeled cholesterol, indicating that TRIM25 promotes cholesterol biosynthesis. Overexpression of INSIG1 partially reversed the effect of TRIM25, reducing the incorporation of the ^13^C label into cholesterol (Figure 5M). However, metabolic flux analysis showed that TRIM25 had no significant effect on fatty acid oxidation (Figure S7, Supporting Information). This confirms that INSIG1 can mitigate the promoting effect of TRIM25 on de novo fatty acid and cholesterol synthesis and further corroborates the mechanism by which TRIM25 negatively regulates INSIG1 protein expression through ubiquitination.

### 2.6 TRIM25 Mediated K11‐ and K48‐Linked Ubiquitination of INSIG1 at Lysines 156 and 158

**Figure 6:**
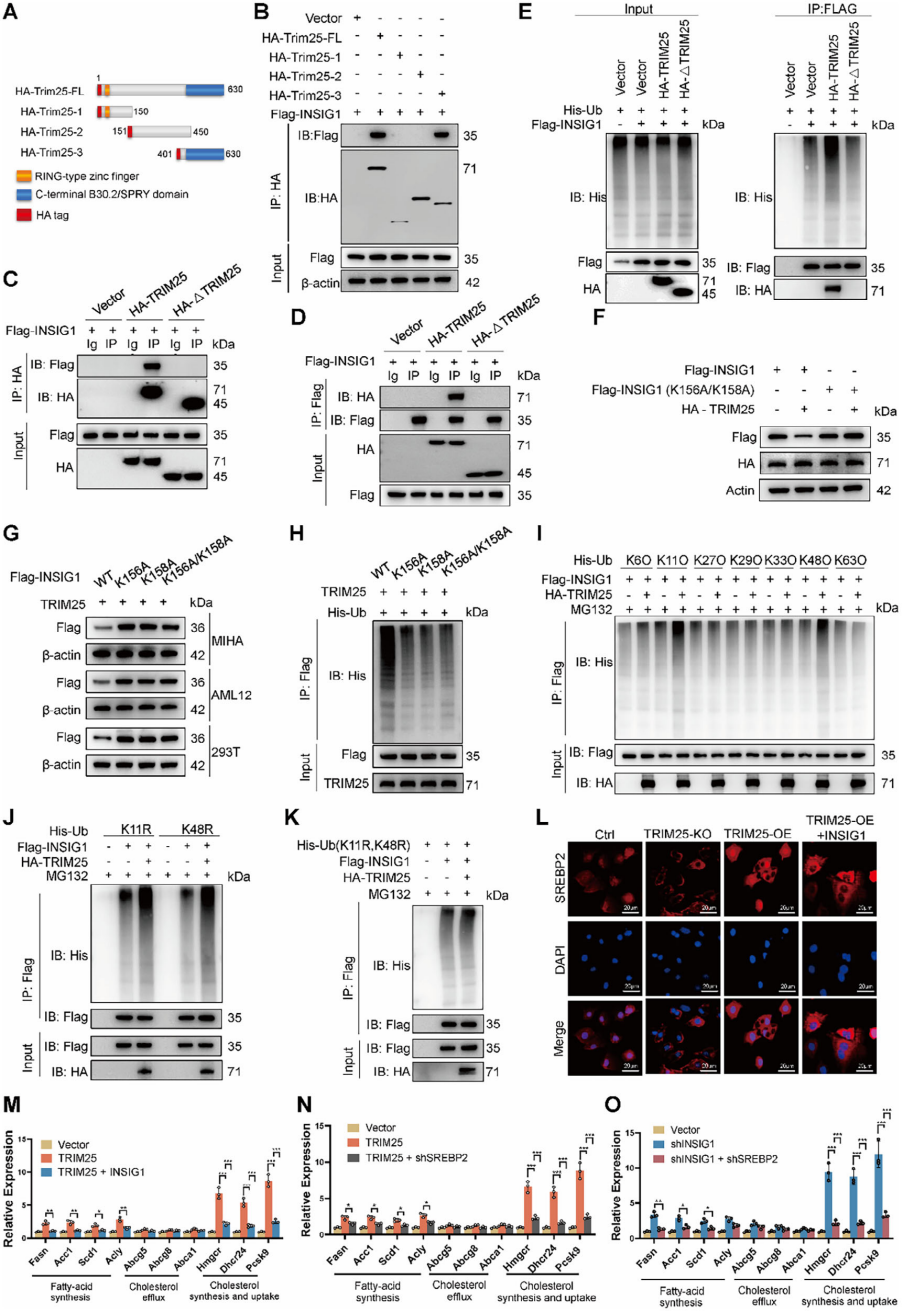
TRIM25 mediates K11‐ and K48‐linked ubiquitination of INSIG1 at lysines 156 and 158. A) TRIM25 truncated plasmids were constructed based on structural features. B) HA‐TRIM25 truncated, and Flag‐INSIG1 plasmids were transfected into MIHA cells, and immunoprecipitation indicated that TRIM25 binds to INSIG1 through its C‐terminal domain (401‐630aa). C,D) A mutant TRIM25 plasmid (HA‐△TRIM25) lacking amino acids 401–630 was constructed. HA‐△TRIM25 or the control plasmid HA‐TRIM25 were co‐transfected with Flag‐INSIG1 into 293T cells, and immunoprecipitation assays were performed. E) Mutations within the C‐terminal domain abolished TRIM25's ability to ubiquitinate INSIG1. F) Compared to the wild‐type INSIG1 plasmid, co‐transfection of the INSIG1 plasmid with lysine mutations at positions 156 and 158 (K156A/K158A) and TRIM25 into 293T cells revealed that the K156A/K158A mutation rendered the protein expression levels resistant to regulation by TRIM25. G,H) Wild‐type, lysine 156 mutant (K156A), lysine 158 mutant (K158A), or combined lysine 156 and 158 mutant INSIG1 plasmids (K156A/K158A) were co‐transfected with TRIM25 into 293T cells. The results indicated that single or combined lysine mutations at positions 156 and 158 in INSIG1 could render the protein and ubiquitination levels of INSIG1 resistant to TRIM25 regulation. I) Co‐transfection with Flag‐INSIG1 and His‐tagged ubiquitin constructs (K6O, K11O, K27O, K29O, K33O, K48O, and K63O) was performed in MIHA cells, followed by overexpression of HA‐TRIM25 to assess the ubiquitination level of INSIG1. The K48O construct is ubiquitin, with all lysines mutated except for K48. J) 293T cells were transfected with His‐tagged K11 mutant (K11R) or K48 mutant (K48R) ubiquitin‐expression plasmids. Cells were pretreated with MG132 for 1 h, and cell lysates were subjected to IP with a Flag antibody to detect ubiquitinated INSIG1. K) The double mutant (K11R/K48R) ubiquitin abolished TRIM25's regulation of INSIG1 ubiquitination. L) Immunofluorescence demonstrated that TRIM25 overexpression promoted SREBP2 nuclear translocation, but this effect was reversed by INSIG1 overexpression. M) In MIHA cells, following overexpression of TRIM25 or simultaneous overexpression of TRIM25 and INSIG1, the expression of lipid metabolism‐related genes was assessed using RT‐qPCR. N) The mRNA levels of lipid metabolism‐related genes were assessed in MIHA cells following TRIM25 overexpression and SREBP1 knockdown. O) In MIHA cells, mRNA levels of lipid metabolism‐related genes were assessed following INSIG1 knockdown or simultaneous knockdown of INSIG1 and SREBP1. Data are presented as mean ± SD. *p < 0.05, **p < 0.01, and ***p < 0.001.

To elucidate the molecular mechanisms of TRIM25‐mediated ubiquitination of INSIG1, including the specific domains, sites, and types of ubiquitination, we constructed various truncated and mutant plasmids. Based on TRIM25's structural features, we generated TRIM25 truncated plasmids (**Figure** **6**A). Immunoprecipitation results indicated that TRIM25 bound to INSIG1 through its C‐terminal domain, encompassing amino acids 401–630 (Figure 6B). Mutations within this region (401–630aa) abolished TRIM25's capacity to bind (Figure 6C,D) and ubiquitinate INSIG1 (Figure 6E). Using the GPS‐Uber online tool, we predicted that INSIG1 ubiquitination occurred at lysine residues K156 and K158. We constructed plasmids with mutations at these sites (K156A, K158A, and the double mutant K156A/K158A). Our results demonstrated that K156A or K158A mutations in INSIG1 render its protein expression resistant to regulation by TRIM25 (Figure 6F,G). Furthermore, K156A or K158A mutations in INSIG1 reduced TRIM25‐mediated ubiquitination of INSIG1 (Figure 6H). To further characterize TRIM25‐mediated ubiquitination of INSIG1, we co‐expressed INSIG1 with mutant ubiquitin isoforms carrying a single lysine residue at positions K6, K11, K27, or other sites (K6O, K11O, K27O, K29O, K33O, K48O, and K63O) in HEK293 cells. This approach enabled us to determine the topology of polyubiquitin chains. Ubiquitination assays revealed that ubiquitin K11O and K48O could ubiquitinate INSIG1. Mutations at K11 or K48 (K11R or K48R, respectively) enabled INSIG1 ubiquitination (Figure 6I,J). However, the double mutant (K11R/K48R) abolished TRIM25's regulation of INSIG1 ubiquitination (Figure 6K). These results indicate that polyubiquitin chain linkage to INSIG1 requires lysine residues K11 or K48. TRIM25 binds directly to INSIG1 through its C‐terminal domain and mediates K11‐ or K48‐linked polyubiquitination at lysine residues K156 and K158. Next, we demonstrated that TRIM25 regulated lipid metabolism via the INSIG1/SREBP2 pathway. TRIM25 overexpression promoted SREBP2 nuclear translocation, whereas INSIG1 knockdown reversed the effect of TRIM25 on SREBP2 subcellular localization (Figure 6L). Gene expression analysis revealed that TRIM25‐induced upregulation of lipid metabolism‐related genes could be reversed by INSIG1 knockdown or SREBP2 overexpression (Figure 6M,N). Additionally, the changes in lipid metabolism‐related genes caused by INSIG1 knockdown were reversed by SREBP2 overexpression (Figure 6O). These findings indicate that TRIM25 mediates K11‐ and K48‐linked ubiquitination of INSIG1 at lysines 156 and 158 and that TRIM25 upregulates hepatic lipid metabolism through the INSIG1/SREBP2 pathway in liver cells.

### 2.7 Screening and Validation of C_27_H_26_N_2_O_4_S as a Small Molecule Inhibitor of TRIM25

**Figure 7:**
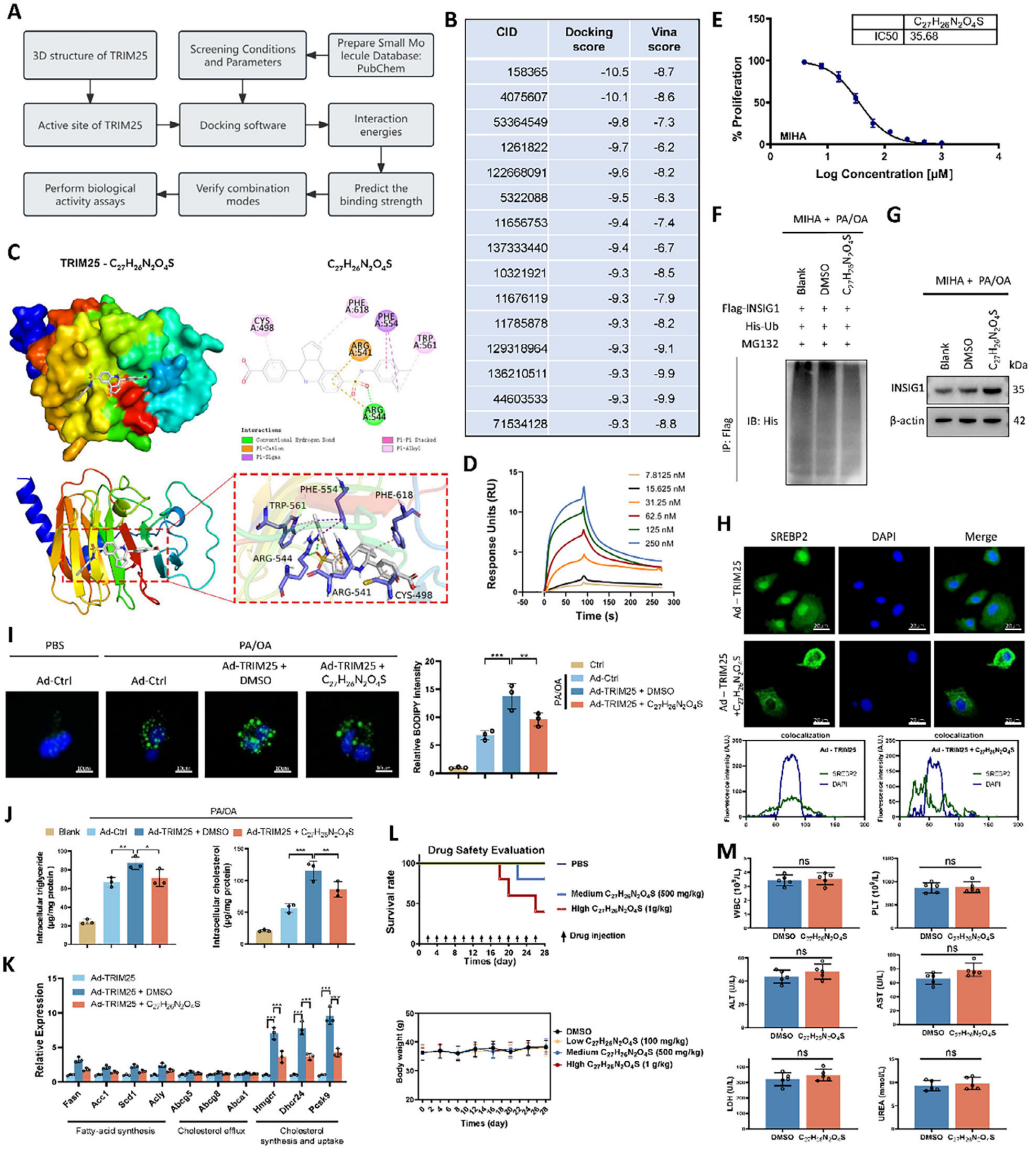
Screening and Validation of C_27_H_26_N_2_O_4_S as a TRIM25 Inhibitor. A) Virtual screening identified potential TRIM25 inhibitors docked into the TRIM25 binding pocket; candidate compounds were selected based on docking scores. B) Top 15 small‐molecule compounds according to docking score. C) A molecular docking model of TRIM25 protein with the small molecule compound C27H26N2O4S was generated using PyMOL software. D) SPR assay confirmed the binding affinity of C_27_H_26_N_2_O_4_S to TRIM25. E) MIHA cells were treated with varying concentrations of C_27_H_26_N_2_O_4_S for 48 h. Cell viability was plotted against drug concentration, and IC_50_ was calculated. F,G) C_27_H_26_N_2_O_4_S treatment in MIHA cells demonstrated decreased INSIG1 ubiquitination (F) and increased INSIG1 protein expression (G) under lipid stress conditions. H) Immunofluorescence of SREBP2 exhibited that the inhibitor reversed TRIM25‐induced SREBP2 nuclear translocation. I) BODIPY fluorescence staining demonstrated that the inhibitor reduced lipid accumulation in cell models. J) The inhibitor C_27_H_26_N_2_O_4_S reduced lipid accumulation in the lipotoxicity model of MIHA cells. K) C_27_H_26_N_2_O_4_S downregulated the mRNA expression levels of genes associated with lipid metabolism. L) After tail vein injection of varying concentrations of C_27_H_26_N_2_O_4_S in mice, the survival status and body weight changes were monitored. M) Following 28 days of tail vein injection of a low concentration of C_27_H_26_N_2_O_4_S (100 mg kg^−1^) in mice, various parameters such as blood routine, liver function, and kidney function were assessed. Data are presented as mean ± SD. *p < 0.05, **p < 0.01, and ***p < 0.001.

To identify novel small‐molecule inhibitors of TRIM25, we conducted virtual screening by docking compounds to the binding pocket of the TRIM25 protein, resulting in a diverse array of compounds (**Figure** **7**A). We selected nine compounds from the top 15 based on their docking scores, with Vina scores lower than –8.0, for experimental validation. We observed that treatment with C_27_H_26_N_2_O_4_S significantly reduced the ubiquitination levels of INSIG1 (Figure 7B; Figure S8A, Supporting Information). Consequently, we selected C_27_H_26_N_2_O_4_S for further analysis. Surface Plasmon Resonance (SPR) assays confirmed the binding of C_27_H_26_N_2_O_4_S to TRIM25 protein (Figure 7C,D). Upon determining the half‐maximal inhibitory concentration (IC_50_) of C_27_H_26_N_2_O_4_S in MIHA cells (Figure 7E), we observed that C_27_H_26_N_2_O_4_S treatment decreased INSIG1 ubiquitination levels and increased INSIG1 protein expression in a lipid toxicity model (Figure 7F,G). Since the binding regions of TRIM25 with either INSIG1 or C_27_H_26_N_2_O_4_S reside within the same domain, we hypothesized that C_27_H_26_N_2_O_4_S might interfere with the interaction between TRIM25 and INSIG1. Immunoprecipitation experiments following C_27_H_26_N_2_O_4_S treatment revealed a significant reduction in the amount of INSIG1 protein bound to TRIM25 (Figure S8B, Supporting Information). Moreover, C_27_H_26_N_2_O_4_S treatment reversed TRIM25‐induced SREBP2 nuclear translocation (Figure 7H). Additionally, C_27_H_26_N_2_O_4_S significantly reduced lipid accumulation in the cellular lipotoxicity model, markedly decreasing intracellular triglyceride and cholesterol levels (Figure 7I,J) and expressing lipid metabolism‐related genes (Figure 7K). Safety assessments indicated that injection of C_27_H_26_N_2_O_4_S at 1 g/kg dose via tail vein did not significantly affect survival rates or liver and kidney functions in C57BL/6 mice (Figure 7L,M; Figure S8C,D, Supporting Information). To confirm the specificity of C_27_H_26_N_2_O_4_S in targeting TRIM25. We constructed mutant plasmids of TRIM25, where the amino acids at positions 498, 541, 544, 554, 561, and 618 were replaced with arginine (TRIM25‐MUT). Both the TRIM25 Wild Type (TRIM25‐WT) and TRIM25‐MUT plasmids resulted in an enhancement of INSIG1 ubiquitination levels, along with an increase in intracellular lipid droplets, triglycerides, and cholesterol. However, C_27_H_26_N_2_O_4_S showed no significant inhibitory effect on the lipid accumulation induced by the TRIM25‐MUT plasmid (Figure S9, Supporting Information). These results collectively demonstrate that C_27_H_26_N_2_O_4_S is a potent inhibitor of TRIM25, capable of modulating INSIG1 ubiquitination and downstream lipid metabolism pathways without apparent toxicity in vivo.

### 2.8 Exosome‐Mediated Encapsulation and Delivery of C_27_H_26_N_2_O_4_S to the Liver

**Figure 8:**
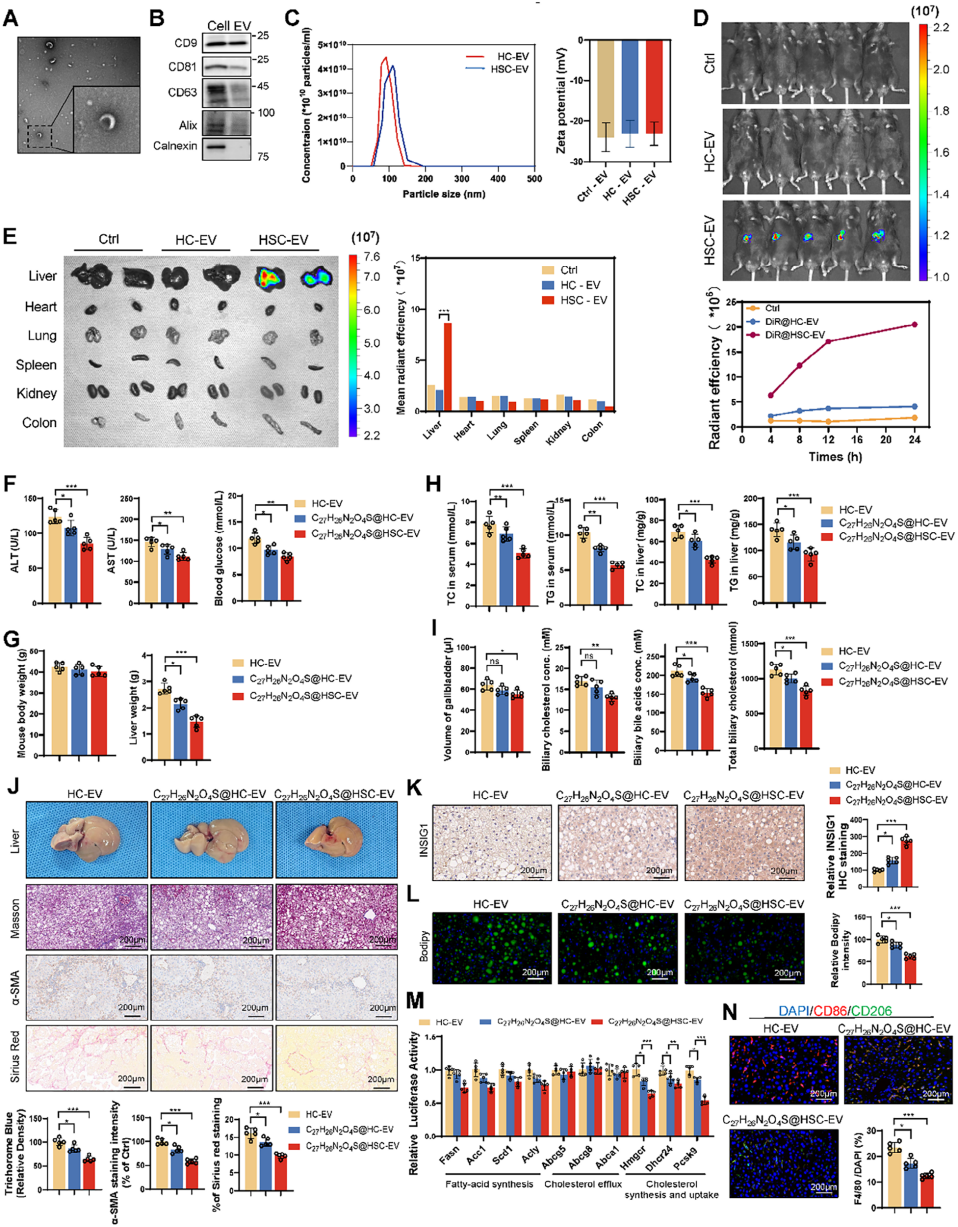
Exosome‐mediated delivery of C_27_H_26_N_2_O_4_S enhances liver targeting and therapeutic efficacy. A) Transmission electron microscopy (TEM) images of HSC‐EVs. B) Protein expression levels of CD9, CD63, CD81, ALIX, and calnexin in the HSC‐derived exosomes. C) Nanosight analysis depicts the particle size distribution of exosomes. D) IVIS imaging of the liver accumulation of DiR‐labeled exosomes after intravenous injection. E) Representative IVIS images of the DiR‐labeled exosomes in different organs, including the heart, lung, kidney, and spleen. F) Administration of C_27_H_26_N_2_O_4_S encapsulated in HSC‐EVs (C_27_H_26_N_2_O_4_S@HSC‐EV) improved liver function in MASH mouse models more effectively than free C_27_H_26_N_2_O_4_S (5 vs 5 vs 5). G,H) C_27_H_26_N_2_O_4_S@HSC‐EV treatment significantly reduced blood glucose, and hepatic lipid content (G), with enhanced therapeutic outcomes compared to non‐encapsulated treatment (H) (5 vs 5 vs 5). I,J) Bile composition analysis and histological examination revealed decreased bile lipids (I) and alleviated hepatic fibrosis (J) in C_27_H_26_N_2_O_4_S@HSC‐EV‐treated mice (5 vs 5 vs 5). K,L) Liver tissues were obtained from mice subjected to HC‐EV, C_27_H_26_N_2_O_4_S@HC‐EV, and C_27_H_26_N_2_O_4_S@HSC‐EV tail vein injections. Immunohistochemical staining for INSIG1 was performed to determine INSIG1 protein levels (K), and BODIPY fluorescence staining was used to evaluate lipid droplet content (L). M) RT‐qPCR was used to detect the expression of lipid metabolism‐related genes in the livers of mice from different groups. N) Immunohistochemical analysis indicated a reduction in the number of macrophage infiltrates, a decrease in the proportion of CD86‐positive M1 macrophages, and an increase in the proportion of CD206‐positive M2 macrophages following C_27_H_26_N_2_O_4_S@HSC‐EV treatment (IVIS: In Vivo Imaging System). *p < 0.05, **p < 0.01, and ***p < 0.001.

The small‐molecule compound C_27_H_26_N_2_O_4_S lacks organ‐targeting specificity, limiting its accumulation in the liver and potentially reducing its therapeutic efficacy while increasing the risk of complications. To address this limitation, we used exosome encapsulation for targeted hepatic delivery. Previous studies have demonstrated that exosomes derived from hepatic stellate cells (HSC‐EVs) significantly accumulate in the liver when injected into mice.^[^ ^28^ ^]^ Consequently, we isolated exosomes from hepatocytes (HC‐EVs) and HSC‐EVs (**Figure** **8**A). The identification of exosomes was accomplished through WB and particle tracking analysis (Figure 8B,C). Exosomes were administered to C57BL/6 mice, and biodistribution analysis confirmed that HSC‐EVs significantly accumulated in the liver compared to other organs, while HC‐EVs displayed no such specificity, Exosomes derived from HEK293 cells were used as a negative control (Ctrl, Cat. C3801, Beyotime) (Figure 8D,E). This finding indicates that HSC‐EVs are more effective for liver targeting. We then encapsulated C_27_H_26_N_2_O_4_S in HSC‐EVs (denoted as C_27_H_26_N_2_O_4_S@HSC‐EV) or HC‐EVs (denoted as C_27_H_26_N_2_O_4_S@HC‐EV) and administered it to a mouse model of MASH via tail vain injection. HC‐EV were used as a negative control. Compared to C_27_H_26_N_2_O_4_S@HC‐EV, C_27_H_26_N_2_O_4_S@HSC‐EV resulted in a more significant improvement in liver function in MASH mice (Figure 8F). Specifically, C_27_H_26_N_2_O_4_S@HC‐EV treatment reduced liver weight/body weight ratio, triglyceride and cholesterol contents in the liver tissue and serum. These effects were more pronounced with C_27_H_26_N_2_O_4_S@HSC‐EV, indicating an enhanced therapeutic efficacy (Figure 8G,H). Additionally, bile component analysis, reflecting the hepatic lipid metabolic state, indicated that C_27_H_26_N_2_O_4_S@HSC‐EV significantly reduced the lipid content, particularly cholesterol, in bile. This observation suggests that C_27_H_26_N_2_O_4_S@HSC‐EV may decrease cholesterol synthesis or promote its excretion from the liver (Figure 8I). Histological examination of liver specimens demonstrated that C_27_H_26_N_2_O_4_S@HSC‐EV significantly alleviated the severity of MASH and hepatic fibrosis in the treated mice (Figure 8J). Compared to the control group, C_27_H_26_N_2_O_4_S@HSC‐EV increased INSIG1 protein levels in liver tissue (Figure 8K), significantly reduced lipid accumulation, and the expression of lipid metabolism‐related genes (Figure 8L,M). Moreover, C_27_H_26_N_2_O_4_S@HSC‐EV treatment significantly decreased the number of macrophages and the proportion of CD86‐positive M1 macrophages in liver tissue (Figure 8N). These results demonstrate that exosome encapsulation, particularly using HSC‐EVs, can effectively target C_27_H_26_N_2_O_4_S in the liver. This targeted delivery enhances the therapeutic effects of the drug in treating MASH, reduces hepatic lipid accumulation, and minimizes the complications associated with systemic distribution.

## 3 Discussion

This study elucidates the pivotal role of TRIM25 in MASH progression and highlights the therapeutic potential of targeting TRIM25 ubiquitin ligase activity. Our multi‐omics analysis revealed a significant TRIM25 upregulation in MASH liver tissues, correlating with disease severity. Functional studies confirmed that TRIM25 promotes lipid accumulation and inflammation in hepatocytes by ubiquitinating and degrading INSIG1, facilitating the nuclear translocation of SREBP2, a master regulator of lipogenesis. These findings suggest that TRIM25‐mediated ubiquitination of INSIG1 is a crucial step in activating SREBP2 and subsequent lipid biosynthesis, contributing to the metabolic disturbances that are characteristic of MASH.

SREBPs have been identified as a transcription factor family that regulates genes involved in lipid synthesis and uptake pathways. SREBPs exist as three isoforms: SREBP‐1a, SREBP‐1c, and SREBP‐2. SREBP‐1c controls the expression of genes involved in fatty acid synthesis, while SREBP‐2 regulates genes associated with cholesterol metabolism.^[^ ^29^, ^30^, ^31^, ^32^ ^]^ The inactive precursor of SREBPs in the endoplasmic reticulum (ER) forms a complex with SREBP cleavage‐activating protein (SCAP) and INSIGs.^[^ ^33^ ^]^ When cholesterol levels in the ER are low, INSIGs are ubiquitinated and degraded, leading to dissociation of the SCAP‐SREBP complex and translocation of the SREBPs precursor to the Golgi apparatus.^[^ ^34^ ^]^ In the Golgi apparatus, precursor SREBPs undergo proteolytic cleavage to become transcriptionally active and subsequently enter the nucleus to regulate the expression of downstream lipid metabolism genes.^[^ ^35^, ^36^ ^]^ INSIG1 and INSIG2 are ER‐resident membrane proteins that block the transport of the SREBP‐SCAP complex from the ER to the Golgi apparatus and accelerate the degradation of HMG‐CoA reductase, thereby reducing cholesterol synthesis.^[^ ^37^ ^]^ Theoretically, an increase in cholesterol intake due to an HFHC diet would enhance the stability of INSIGs, leading to a decrease in cholesterol synthesis. However, our study found that upregulation of TRIM25 expression in MASH resulted in ubiquitination and degradation of INSIG1, thereby enhancing nuclear translocation of SREBPs and lipid synthesis. To determine whether TRIM25 overexpression promotes lipid accumulation in other tissue cells, we selected the LX‐2, HPDE6‐C7, HIEC‐6, and BEAS‐2B cell lines. The experimental results indicated that overexpression of TRIM25 in these cell lines also led to an increase in the ubiquitination levels of INSIG1. However, it is regrettable that in these cell lines, we did not observe a significant increase in lipid accumulation. This may be attributed to the fact that the de novo lipid synthesis processes in these tissues are not as active as that in hepatocytes (Figure S10, Supporting Information).

INSIG1 functions as an inhibitory factor in regulating SREBPs by preventing their transport to the Golgi apparatus and the subsequent proteolytic processing in the ER. INSIG1 is regulated by ubiquitination and degradation mediated by GP78.^[^ ^38^ ^]^ However, little is known about how INSIG1 protein in hepatocytes is regulated under physiological and pathological conditions. This study provides compelling evidence that TRIM25 is an upstream regulatory factor for INSIG1 in hepatocytes. TRIM25 directly interacts with INSIG1 and promotes its ubiquitination and degradation. This, in turn, enhances the activation and nuclear translocation of SREBPs, ultimately leading to NASLD occurrence and progression. Finally, we corroborated the conclusions of our study through rescue experiments. Following INSIG1 knockdown, we observed a pronounced increase in the expression of lipid synthesis‐related genes, as well as intracellular triglyceride and cholesterol levels. However, in INSIG1‐deficient MIHA cells, further knockdown of TRIM25 did not lead to the marked suppression of lipid synthesis (Figure S11, Supporting Information). These results indicate that TRIM25 mediates its effects on lipid synthesis through INSIG1. We have demonstrated in our research that TRIM25 can increase immune cell infiltration in the livers of MASH mice. However, does TRIM25 directly regulate the immune inflammatory response, or does it induce lipotoxicity through enhanced lipid accumulation, subsequently triggering the immune response? To address this question, we utilized adeno‐associated virus (AAV) to overexpress TRIM25 in the mouse liver and assessed the immune cells present. After injecting AAV‐Ctrl and AAV‐TRIM25, the NCD mice were kept for four weeks, and their livers were collected for immune flow cytometry analysis. The results revealed that, compared to AAV‐Ctrl, the lipid content in the livers of mice injected with AAV‐TRIM25 showed a notable increase, yet the macrophage content did not exhibit significant changes (Figure S12, Supporting Information).

Using exosomes for targeted drug delivery to specific organs or tissues is a promising strategy for therapeutic development. Various methods exist for directing exosomes to the liver, such as engineering exosomes through genetic and chemical methods for targeted drug delivery.^[^ ^39^, ^40^ ^]^ In our study, we opted for HSC‐EVs to deliver C_27_H_26_N_2_O_4_S, demonstrating efficient hepatic accumulation and high therapeutic efficacy in animal models.^[^ ^28^ ^]^ However, a limitation of our research is the lack of comparative analysis with other delivery methods. Future studies should explore alternative drug delivery strategies to optimize therapeutic outcomes.^[^ ^41^ ^]^

Our findings demonstrate that TRIM25 is a crucial regulator of lipid metabolism and inflammation in MASH. Inhibition of TRIM25 by C_27_H_26_N_2_O_4_S, exclusively when delivered via HSC‐EVs, presents a promising therapeutic approach for MASH. This study provides new insights into the molecular mechanisms driving MASH progression and underscores the potential of targeting ubiquitination pathways for treating metabolic liver diseases. Future research should focus on optimizing the delivery and efficacy of TRIM25 inhibitors and exploring their potential in clinical settings.

## 4 Experimental Section

### Cell Culture

MIHA, AML12, and HEK293T cell lines were procured from the American Type Culture Collection. All cell lines were grown in Dulbecco's Modified Eagle Medium with 10% fetal bovine serum and 1% penicillin/streptomycin. Cells were subcultured every 2–3 days to ensure logarithmic growth and were used for experiments at 70–80% confluence. All procedures were conducted under aseptic conditions within a biological safety cabinet.

### Patients and Samples

Human liver tissues were obtained from the hepatobiliary surgery department of the Second Hospital of Shandong University. The informed consent forms were obtained from patients and their families. The clinical patient specimen experiments involved in this study were authorized and approved by the Research Ethics Committee of the Second Hospital of Shandong University (KYLL‐2023‐413).

### Real‐Time Quantitative PCR (RT‐qPCR)

Total RNA was isolated with the TRIzol reagent (Invitrogen, Carlsbad, CA) as previously described.^[^ ^42^ ^]^ The primer sequences for the target genes and *β*‐actin are detailed in Table S1 (Supporting Information).

### WB Analysis

WB was performed as previously described.^[^ ^42^ ^]^ Membranes were incubated with primary antibodies (Table S2, Supporting Information). Bands were detected using ECL and quantified with ImageJ. Data were normalized to internal controls and analyzed statistically.

### Detection of IL‐6 and TNF‐*α* Levels

The concentrations of IL‐6 and TNF‐*α* in the cell culture supernatants were quantified using enzyme‐linked immunosorbent assay (ELISA) kits. Specifically, the Human IL‐6 ELISA Kit and Human TNF‐*α* ELISA Kit were utilized, both purchased from LiankeBio (China). The assays were performed according to the manufacturer's instructions.

### Animal Model

Male C57BL/6 mice, aged 8–10 weeks, were procured from Shanghai Model Organism Center, Inc. To develop MASH models, mice were randomly assigned to two groups and fed either an HFD or an HFHC diet for 24 weeks. For the MASLD mouse model, a 12‐week feeding period with either the HFD or HFHC diet was sufficient. The HFD contained 60% of calories from fat, while the HFHC diet included an additional 1.25% cholesterol. This diet was selected based on previous studies demonstrating its efficacy in inducing MASLD and MASH in C57BL/6 mice. A control group of C57BL/6 mice was maintained on a standard chow diet (10% of calories from fat) throughout the experimental period to serve as a baseline for comparison. The male C57BL/6 mice were raised in the SPF animal room of the Animal Center of the Second Hospital of Shandong University. All animal experiments were performed following the ARRIVE reporting guidelines. This study was approved by the Second Hospital of Shandong University (KYLL‐2023‐412).

### Histological and Immunohistochemical Staining

Hematoxylin and eosin (H&E) staining was performed using a commercially available staining kit (C0105, Beyotime, China). Briefly, tissues were fixed in 4% paraformaldehyde, dehydrated, embedded in paraffin, and sectioned at a thickness of 4 µm. The sections were deparaffinized with xylene, rehydrated through a graded ethanol series, and stained with hematoxylin for nuclear visualization, followed by eosin for cytoplasmic contrast. Slides were dehydrated, cleared, and mounted for microscopic analysis. For immunohistochemical (IHC) staining, paraffin‐embedded sections were first deparaffinized and rehydrated as described above. Antigen retrieval was performed by incubating the sections in citrate buffer (pH 6.0) at 95 °C for 15 min. Endogenous peroxidase activity was quenched by treating the slides with 3% hydrogen peroxide for 10 min. Non‐specific binding was blocked with 5% bovine serum albumin (BSA) for 30 min at room temperature. Primary antibodies were applied as detailed in Table S2 (Supporting Information). Biotinylated secondary antibodies were applied sequentially, followed by DAB staining.

### Immunofluorescence Staining

Cells were cultured on coverslips until 50–60% confluent. Fixation was performed using 4% paraformaldehyde for 15 min. They were then fixed with 4% paraformaldehyde and 0.1% Triton X‐100 permeabilization, followed by blocking with 5% bovine serum albumin (BSA). Primary antibodies, as detailed in Table S2 (Supporting Information), were diluted and incubated with the cells. The cells were subsequently incubated with secondary antibodies and DAPI. Coverslips were washed, mounted using antifade medium, and sealed. Imaging was conducted using a Zeiss LSM 980 confocal microscope.

### BODIPY Lipid Droplet Staining

Cells were cultured on coverslips to 50%–60% confluency, fixed with 4% paraformaldehyde for 15 min, and incubated with 1 µg mL^−1^ BODIPY 493/503 (MCE, Cat# HY‐W090090) for 30 min. After washing with PBS, nuclei were counterstained with DAPI for 5 min. For tissue staining, mouse liver tissues were harvested, snap‐frozen in liquid nitrogen. The subsequent steps of fixation and staining for the sections were performed in the same manner as the BODIPY staining of the cells described previously.

### Masson's Trichrome Staining

Mouse liver tissues were fixed in 10% formalin, embedded in paraffin, and sectioned at 5 µm thickness. Deparaffinized sections were stained using Masson's Trichrome Stain Kit (Sigma–Aldrich). Sections were sequentially stained with hematoxylin, Biebrich scarlet‐acid fuchsin, and aniline blue. After differentiation and counterstaining, sections were mounted with synthetic resin.

### Sirius Red Staining

Deparaffinized liver sections were stained with Sirius Red (Direct Red 80, Sigma‐Aldrich) in saturated aqueous picric acid for 1 h. Post‐staining sections were washed and mounted using synthetic resin.

### Stable Isotope Tracing Experiments

The cells were cultured in DMEM (0 mmol L^−1^ glucose) for 24 h. Then, the medium was replaced with DMEM containing 10 mmol L^−1^ [13C]‐labeled glucose, and the cells were incubated at 37 °C for an additional 72 h. The cells were washed with PBS, and a mixture of methanol/PBS (1:1, v/v) was added. The cells were then collected, and chloroform was added to the cell suspension for lipid extraction. The mixture was vigorously vortexed and centrifuged to separate the lipid‐containing organic phase. The collected lipids were then hydrolyzed and subjected to liquid chromatography‐mass spectrometry (LC‐MS) for further investigation. Cells were cultured in DMEM containing 2 mM [13C]‐labeled acetate to assess cholesterol synthesis for 72 h. Cells were collected, pelleted, and quickly frozen in liquid nitrogen. Lipids were extracted by adding the cell lysate to 3 mL of CH_3_Cl:CH_3_OH mixture (2:1, v/v). The resulting mixture was vigorously vortexed and centrifuged to separate the organic phase containing lipids.

### Immunoprecipitation and Mass Spectrometry Analysis

Immunoprecipitation and mass spectrometry were performed as previously described.^[^ ^29^ ^]^

### GST‐Pulldown Assay

To demonstrate the direct interaction between TRIM25 and INSIG1, it performed GST‐pulldown assays using the GST‐TRIM25 and GST‐INSIG1 fusion proteins. GST fusion proteins, Flag‐tagged INSIG1 and HA‐tagged TRIM25 proteins were expressed in *Escherichia coli* BL21 (DE3) cells. The plasmid expressing the fusion protein was transformed into competent E. coli BL21 (DE3) cells (TransGen Biotech, China) using the heat‐shock method. Positive transformants were selected on LB agar plates containing 50 µg mL^−1^ of kanamycin. A single colony was then inoculated into 10 mL of LB medium supplemented with kanamycin (50 µg mL^−1^) and cultivated overnight at 37 °C with shaking at 200 rpm. For the purification of Flag‐tagged, His‐tagged, or GST‐tagged fusion proteins, the corresponding Thermo Fisher magnetic agarose resin kits were utilized (Catalog #88836, #A36801 and #25236). The choice of magnetic resin depended on the specific tag fused to the target protein. The detailed protocol for each type of resin was followed according to the manufacturer's instructions with minor modifications. In the first set of experiments, purified GST‐TRIM25 or GST alone (control) was incubated with purified Flag‐tagged INSIG1. Bound proteins were eluted by boiling the beads in SDS sample buffer and were analyzed by SDS‐PAGE with an anti‐Flag antibody to detect INSIG1. In the second set of experiments, purified GST‐INSIG1 or GST alone was incubated with purified HA‐TRIM25 under the same conditions. Following incubation, washing, and elution, the proteins were analyzed by SDS‐PAGE with an anti‐HA antibody to detect TRIM25.

### Subcellular Fractionation Assay

To investigate the distribution of proteins between the nuclear and cytoplasmic compartments, the separation of nucleus and cytoplasm in MIHA cell lines was performed following the manufacturer's instructions of NE‐PER Nuclear and Cytoplasmic Extraction Reagents (Thermo Fisher), utilizing tubulin and histone H3 as reference markers for the cytoplasmic and nuclear fractions, respectively.

### Ubiquitination Assay

The ubiquitination assay was performed as previously described.^[^ ^43^ ^]^ Initially, the cells were transfected with His‐ubiquitin and other plasmids. After 24 h, cells were treated with the proteasome inhibitor MG132 (Sigma, M7449) at a concentration of 25 mM for 4 h. The cells were then lysed in a buffer and incubated overnight with an anti‐INSIG1 antibody. Finally, the samples were separated by SDS‐PAGE and analyzed by WB. Ubiquitinated INSIG1 was detected using an anti‐His antibody.

### Exosome Extraction

The cells were cultured in their respective media until 80% confluency was reached. The media was collected and centrifuged at 300 × *g* for 10 min. The supernatant, containing the exosomes, was further centrifuged at 2000 × *g* for 20 min at 4 °C. The clarified supernatant was subjected to ultracentrifugation at 100000 × *g* for 70 min at 4 °C using a SW41Ti rotor (Beckman Coulter). The resulting pellet, enriched with exosomes, was resuspended in PBS and stored at −80 °C until further analysis. To confirm the presence of exosomes, it performed nanoparticle tracking analysis using a NanoSight NS300 (Malvern Instruments) to visualize and quantify the exosomes based on their size and concentration. Additionally, exosome markers, such as CD63 and CD81, were assessed by WB analysis of the isolated exosomes to ensure their purity and integrity.

### In Vivo Imaging of Exosome

To investigate the distribution and dynamics of exosomes in vivo, it used DiR‐labeled exosomes for imaging. Briefly, exosomes were incubated with DiR for 30 min at 37 °C, followed by ultracentrifugation. The labeled exosomes were resuspended in PBS. Mice were intravenously injected with DiR‐labeled exosome (DiR@HC‐EV, and DiR@HSC‐EV), fluorescence dye DiR was taken as control. The final concentration of *DIR* was 100 µg mL^−1^ in each formulation. C57BL/c mice received a tail vein injection of 100 µL of fluorescence dye DiR and DiR‐labeled exosomes (DiR@HSC‐EV or DiR@HC‐EV, 10 µg *DIR* of each mouse). Mice were anesthetized with isoflurane, and images were taken using the IVIS Spectrum system (Caliper, USA) at 4, 8, 12, and 24‐h post‐injection. The mice were euthanized, and the major organs were harvested for ex vivo imaging.

### Encapsulation of Small Molecule C_27_H_26_N_2_O_4_S into Exosomes

To encapsulate the small‐molecule C_27_H_26_N_2_O_4_S into exosomes, it used an electroporation method. Exosomes were isolated from hepatocytes and hepatic stellate cells using ultracentrifugation as previously described. The purified exosomes were resuspended in an electroporation buffer (PBS with 200 mM sucrose). A solution containing 1 mM of C_27_H_26_N_2_O_4_S was prepared and mixed with the exosome suspension. The mixture was placed in an electroporation cuvette and subjected to a single electrical pulse using a Gene Pulser Xcell Electroporation System (Bio‐Rad) set at 400 V and 125 µF. Following electroporation, the exosome mixture was incubated on ice for 30 min to allow membrane recovery. The mixture was subjected to another round of ultracentrifugation at 100000 × *g* for 70 min at 4 °C to remove free small molecules not encapsulated within the exosomes.

### Statistical Analysis

Statistical analyses were performed using GraphPad Prism software (version 8.0; GraphPad Software, San Diego, CA). Data were presented as mean ± standard deviation (SD) from at least three independent experiments unless otherwise specified. The sample size (n) for each experiment was indicated in the corresponding figure legend. Comparisons between two groups were performed using a two‐tailed Student's t‐test for normally distributed data. For comparisons involving more than two groups, a one‐way analysis of variance followed by Tukey's post‐hoc test was used for normally distributed data. A *p* < 0.05 was considered statistically significant. The following thresholds were used to denote statistical significance: *p < 0.05, **p < 0.01, and ***p < 0.001.

## Conflict of Interest

The authors declare no conflict of interest.

## Author Contributions

H.Z., X.K., and W.W. contributed equally to this work. The manuscript was written through contributions of all authors. H.Z., W.W., X.Z. and B.J. designed the experiments. H.Z., X.K., W.W. and Z.G. performed all the experiments. H.Z., Z.G. and H.W. analyzed the data. H.Z. and H.Q. write original draft. All authors have given approval to the final version of the manuscript.

## Supporting information



Supporting Information

## Data Availability

The data that support the findings of this study are available from the corresponding author upon reasonable request.

## References

[1] M. E. Rinella , J. V. Lazarus , V. Ratziu , S. M. Francque , A. J. Sanyal , F. Kanwal , D. Romero , M. F. Abdelmalek , Q. M. Anstee , J. P. Arab , M. Arrese , R. Bataller , U. Beuers , J. Boursier , E. Bugianesi , C. D. Byrne , G. E. Castro Narro , A. Chowdhury , H. Cortez‐Pinto , D. R. Cryer , K. Cusi , M. El‐Kassas , S. Klein , W. Eskridge , J. Fan , S. Gawrieh , C. D. Guy , S. A. Harrison , S. U.p Kim , B. G. Koot , J. Hepatol. 2023, 79, 1542. 37364790 10.1016/j.jhep.2023.06.003

[2] M. E. Rinella , J. V. Lazarus , V. Ratziu , S. M. Francque , A. J. Sanyal , F. Kanwal , D. Romero , M. F. Abdelmalek , Q. M. Anstee , J. P. Arab , M. Arrese , R. Bataller , U. Beuers , J. Boursier , E. Bugianesi , C. D. Byrne , G. E. Castro Narro , A. Chowdhury , H. Cortez‐Pinto , D. R. Cryer , K. Cusi , M. El‐Kassas , S. Klein , W. Eskridge , J. Fan , S. Gawrieh , C. D. Guy , S. A. Harrison , S. U.p Kim , Hepatology. 2023, 78, 1966.37363821

[3] G. Targher , C. D. Byrne , H. Tilg , Gut. 2024, 73, 691. 38228377 10.1136/gutjnl-2023-330595

[4] J. M. Pericas , Q. M. Anstee , S. Augustin , R. Bataller , A. Berzigotti , A. Ciudin , S. Francque , J. G. Abraldes , V. Hernandez‐Gea , M. Pons , T. Reiberger , I. A. Rowe , P. Rydqvist , E. Schabel , F. Tacke , E. A. Tsochatzis , J. Genesca , Nat. Rev. Gastroenterol. Hepatol. 2024, 21, 809. 39020089 10.1038/s41575-024-00955-8

[5] V. Taru , G. Szabo , W. Mehal , T. Reiberger , J. Hepatol. 2024, 81, 895. 38908436 10.1016/j.jhep.2024.06.016 PMC11881887

[6] Z. Younossi , Q. M. Anstee , M. Marietti , T. Hardy , L. Henry , M. Eslam , J. George , E. Bugianesi , Nat. Rev. Gastroenterol. Hepatol. 2018, 15, 11. 28930295 10.1038/nrgastro.2017.109

[7] Z. M. Younossi , P. Golabi , J. M. Paik , A. Henry , C. Van Dongen , L. Henry , Hepatology. 2023, 77, 1335. 36626630 10.1097/HEP.0000000000000004 PMC10026948

[8] R. H. Friedline , H. L. Noh , S. Suk , M. Albusharif , S. Dagdeviren , S. Saengnipanthkul , B. Kim , A. M. Kim , L. H. Kim , L. A. Tauer , N. M. Baez Torres , S. Choi , B.‐Y. Kim , S. D. Rao , K. Kasina , C. Sun , B. J. Toles , C. Zhou , Z. Li , V. M. Benoit , P. R. Patel , D. X. T. Zheng , K. Inashima , A. Beaverson , X. Hu , D. A. Tran , W. Muller , D. L. Greiner , A. C. Mullen , K. W. Lee , Nat. Commun. 2024, 15, 5506. 38951527 10.1038/s41467-024-49633-y PMC11217362

[9] S. Hu , R. Li , D. Gong , P. Hu , J. Xu , Y. Ai , X. Zhao , C. Hu , M. Xu , C. Liu , S. Chen , J. Fan , Z. Zhao , Z. Zhang , H. Wu , Y. Xu , Sci. Adv. 2024, 10, ado3141. 10.1126/sciadv.ado3141 PMC11268416 39047111

[10] Y. Kwon , P. Gottmann , S. Wang , J. Tissink , K. Motzler , R. Sekar , W. Albrecht , C. Cadenas , J. G. Hengstler , A. Schurmann , A. Zeigerer , J. Hepatol. 2024, 82, 18. 38977136 10.1016/j.jhep.2024.06.040

[11] Y. Li , L. Chen , C. Sottas , M. C. Raul , N. D. Patel , J. R. Bijja , S. K. Ahmed , A. Kapelanski‐Lamoureux , A. Lazaris , P. Metrakos , A. Zambidis , S. Chopra , M. Li , G.o Sugahara , T. Saito , V. Papadopoulos , Metabolism. 2024, 159, 155942. 38871077 10.1016/j.metabol.2024.155942 PMC11374472

[12] Z. Li , D. Zheng , T. Zhang , S. Ruan , N. Li , Y. Yu , Y. Peng , D. Wang , Hepatol. Commun. 2024, 8, 0343. 10.1097/HC9.0000000000000343 PMC10727660 38099854

[13] H. Zhang , P. Xia , Z. Yang , J. Liu , Y. Zhu , Z. Huang , Z. Zhang , Y. Yuan , Clin. Transl. Med. 2023, 13, 1443. 10.1002/ctm2.1443 PMC10576442 37837399

[14] A. Das , H. Cheng , Y. Wang , L. N. Kinch , G. Liang , S. Hong , H. H. Hobbs , J. C. Cohen , Proc. Natl. Acad. Sci. USA 2024, 121, 2312291121. 10.1073/pnas.2312291121 PMC10861911 38294943

[15] M.‐C. Xiao , N. Jiang , L.‐L. Chen , F. Liu , S.‐Q. Liu , C.‐H. Ding , S.‐H. Wu , K.‐Q. Wang , Y.‐Y. Luo , Y. Peng , F.‐Z. Yan , X. Zhang , H. Qian , W.‐F. Xie , J Hepatol. 2024, 80, 778. 38237865 10.1016/j.jhep.2023.12.029

[16] S. Xu , X. Wu , S. Wang , M. Xu , T. Fang , X. Ma , M. Chen , J. Fu , J. Guo , S. Tian , T. Tian , X. Cheng , H. Yang , J. Zhou , Z. Wang , Y. Yin , W. Xu , F. Xu , J. Yan , Z. Wang , S. Luo , X.‐J. Zhang , Y.‐X. Ji , J. Weng , J. Clin. Invest. 2024, 134. 10.1172/JCI166149 PMC10904058 38206764

[17] Y. Chen , X. Xu , K. Ding , T. Tang , F. Cai , H. Zhang , Z. Chen , Y. Qi , Z. Fu , G. Zhu , Z. Dou , J. Xu , G. Chen , Q. Wu , J. Ji , J. Zhang , J Exp Clin Cancer Res. 2024, 43, 39. 38303029 10.1186/s13046-024-02964-6 PMC10835844

[18] Z. Shang , S. Zhang , J. Wang , L. Zhou , X. Zhang , D. D. Billadeau , P. Yang , L. Zhang , F. Zhou , P. Bai , D. Jia , Nat. Commun. 2024, 15, 4127. 38750080 10.1038/s41467-024-48596-4 PMC11096359

[19] K.‐I. Takayama , T. Suzuki , T. Tanaka , T. Fujimura , S. Takahashi , T. Urano , K. Ikeda , S. Inoue , Oncogene. 2018, 37, 2165. 29379164 10.1038/s41388-017-0095-x

[20] Y. Zou , S. Wu , Q. Hu , H. Zhou , Y. Ge , Z. Ju , S. Luo , J. Adv. Res. 2024, 68, 387. 38479571 10.1016/j.jare.2024.03.006 PMC11785578

[21] Y. P. Bai , T. Zhang , Z. Y. Hu , Y. Zhang , D. G. Wang , M. Y. Zhou , Y. Zhang , F. Zhang , X. Kong , Biochem. Pharmacol. 2024, 224, 116240. 38679210 10.1016/j.bcp.2024.116240

[22] C. L. Horn , A. L. Morales , C. Savard , G. C. Farrell , G. N. Ioannou , Hepatol Commun. 2022, 6, 12. 34558856 10.1002/hep4.1801 PMC8710790

[23] S. Xie , L. Yuan , Y. Sui , S. Feng , H. Li , X. Li , EMBO Rep. 2024, 25, 378. 38177901 10.1038/s44319-023-00012-6 PMC10897415

[24] S. Hendrix , J. Kingma , R. Ottenhoff , M. Valiloo , M. Svecla , L. F. Zijlstra , V. Sachdev , K. Kovac , J. H. M. Levels , A. Jongejan , J. F. de Boer , F. Kuipers , A. Rimbert , G. D. Norata , A. Loregger , N. Zelcer , Nat. Commun. 2023, 14, 5181. 37626055 10.1038/s41467-023-40943-1 PMC10457316

[25] S. Vaidyanathan , T. M. Salmi , R. M. Sathiqu , M. J. McConville , A. G. Cox , K. K. Brown , Dev. Cell. 2022, 57, 719. 35216681 10.1016/j.devcel.2022.02.004

[26] C. Cheng , F. Geng , Z. Li , Y. Zhong , H. Wang , X. Cheng , Y. Zhao , X. Mo , C. Horbinski , W. Duan , A. Chakravarti , X. Cheng , D. Guo , Nat Metab. 2022, 4, 575. 35534729 10.1038/s42255-022-00568-y PMC9177652

[27] Y. Wang , Y. Wang , L. Ding , X. Ren , B. Wang , L. Wang , S. Zhao , X. Yue , Z. Wu , C. Li , X. Liang , C. Ma , L. Gao , Cell Rep. 2022, 41, 111738. 36450259 10.1016/j.celrep.2022.111738

[28] T. Wan , J. Zhong , Q. Pan , T. Zhou , Y. Ping , X. Liu , Sci. Adv. 2022, 8, abp9435. 10.1126/sciadv.abp9435 PMC9473578 36103526

[29] P. Xia , H. Zhang , H. Lu , K. Xu , X. Jiang , Y. Jiang , X. Gongye , Z. Chen , J. Liu , X. Chen , W. Ma , Z. Zhang , Y. Yuan , Cancer Commun. 2023, 43, 338. 10.1002/cac2.12403 PMC10009668 36602428

[30] H. Zeng , H. Qin , M. Liao , E. Zheng , X. Luo , A. Xiao , Y. Li , L. Chen , L. Wei , L. Zhao , X. Z. Ruan , P. Yang , Y. Chen , Mol. Metab. 2022, 57, 101428. 34974159 10.1016/j.molmet.2021.101428 PMC8810570

[31] Y. Benatzy , M. A. Palmer , D. Lütjohann , R.‐I. Ohno , N. Kampschulte , N. H. Schebb , D. C. Fuhrmann , R. G. Snodgrass , B. Brüne , Redox Biol. 2024, 72, 103149. 38581859 10.1016/j.redox.2024.103149 PMC11002893

[32] E. H. K. Mok , C. O. N. Leung , L. Zhou , M. M. L. Lei , H. W. Leung , M. Tong , T. L. Wong , E. Y. T. Lau , I. O. L. Ng , J. Ding , J. P. Yun , J. Yu , H. L. Zhu , C. H. Lin , D. Lindholm , K. S. Leung , J. D. Cybulski , D. M. Baker , S. Ma , T. K. W. Lee , Cancer Res. 2022, 82, 3102. 35767704 10.1158/0008-5472.CAN-21-2934

[33] M. S. Brown , J. L. Goldstein , Cell. 1997, 89, 331.9150132

[34] H. Shimano , R. Sato , Nat. Rev. Endocrinol. 2017, 13, 710. 28849786 10.1038/nrendo.2017.91

[35] M. S. Brown , A. Radhakrishnan , J. L. Goldstein , Annu. Rev. Biochem. 2018, 87, 783. 28841344 10.1146/annurev-biochem-062917-011852 PMC5828883

[36] D. L. Kober , A. Radhakrishnan , J. L. Goldstein , M. S. Brown , L. D. Clark , X.‐C. Bai , D. M. Rosenbaum , Cell. 2021, 184, 3689e22. 34139175 10.1016/j.cell.2021.05.019 PMC8277531

[37] R. A. Faulkner , Y. Yang , J. Tsien , T. Qin , R. A. DeBose‐Boyd , Proc. Natl. Acad. Sci. 2024, 121, 2318822121. 10.1073/pnas.2318822121 PMC10873557 38319967

[38] T. Xu , W. Yu , H. Fang , Z. Wang , Z. Chi , X. Guo , D. Jiang , K. Zhang , S. Chen , M. Li , Y. Guo , J. Zhang , D. Yang , Q. Yu , D. Wang , X. Zhang , Cell Death Differ. 2022, 29, 1582. 35110683 10.1038/s41418-022-00947-8 PMC9345978

[39] Y. Liang , L. Duan , J. Lu , J. Xia , Theranostics. 2021, 11, 3183. 33537081 10.7150/thno.52570 PMC7847680

[40] X. Lu , H. Guo , X. Wei , D. Lu , W. Shu , Y. Song , N. Qiu , X. Xu , Int J Nanomedicine. 2023, 18, 2873. 37283714 10.2147/IJN.S404925 PMC10239634

[41] C. Wang , M. Xu , Q. Fan , C. Li , X. Zhou , Asian J Pharm. Sci. 2023, 18, 100772. 36896446 10.1016/j.ajps.2022.100772 PMC9989662

[42] H. Zhang , T. Xia , Z. Xia , H. Zhou , Z. Li , W. Wang , X. Zhai , B. Jin , Cell. Mol. Life Sci. 2024, 81, 96. 38372748 10.1007/s00018-024-05114-5 PMC10876760

[43] H. Zhang , X. Zhai , Y. Liu , Z. Xia , T. Xia , G. Du , H. Zhou , D. Franziska Strohmer , A. V. Bazhin , Z. Li , X. Wang , B. Jin , D. Guo , Research 2023, 6, 0184. 37398932 10.34133/research.0184 PMC10313139

