# Supplementary material for: TRIM25‐Mediated INSIG1 Ubiquitination Promotes MASH Progression Through Reprogramming Lipid Metabolism
Source: Adv Sci (Weinh). 2025 Apr 15;12(21):2414646. doi: 10.1002/advs.202414646 (PMC12140344; doi:10.1002/advs.202414646)
Supplement: Supplementary file 1 — Supporting Information [file ADVS-12-2414646-s001.pdf]

ADVANCED SCIENCE
Open Access

Supporting Information

for *Adv. Sci.*, DOI 10.1002/advs.202414646

TRIM25-Mediated INSIG1 Ubiquitination Promotes MASH Progression Through Reprogramming Lipid Metabolism

Hao Zhang, Xiangxu Kong, Wei Wang, Huaxin Zhou, Haoran Qu, Zhengyao Guan, Honglei Wu, Xiangyu Zhai* and Bin Jin**

Supplementary Figures

Figure S1

A

B

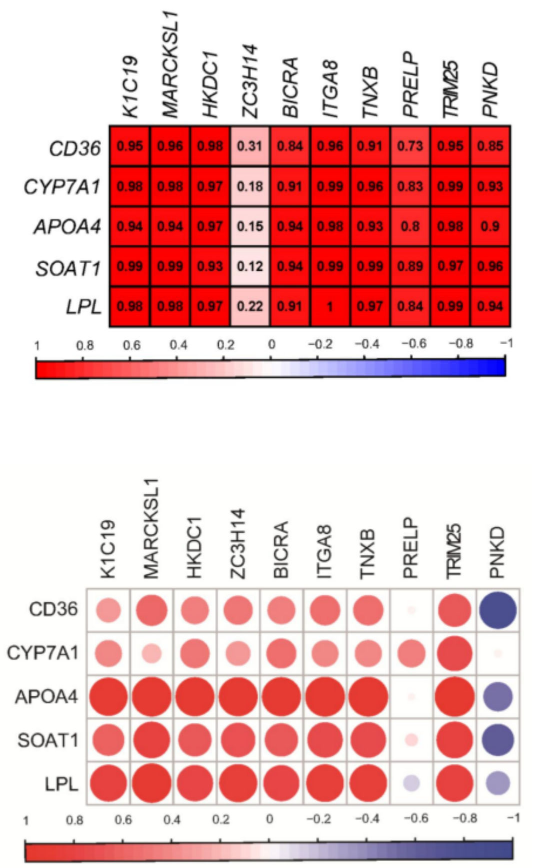

Figure S1. Correlation analysis was performed between genes associated with cholesterol and triglycerides and genes enriched in metabolic pathways. **(A)** Correlation analysis was conducted at the transcriptomic gene level. **(B)** Correlation analysis at the protein level in proteomics sequencing.

Figure S2

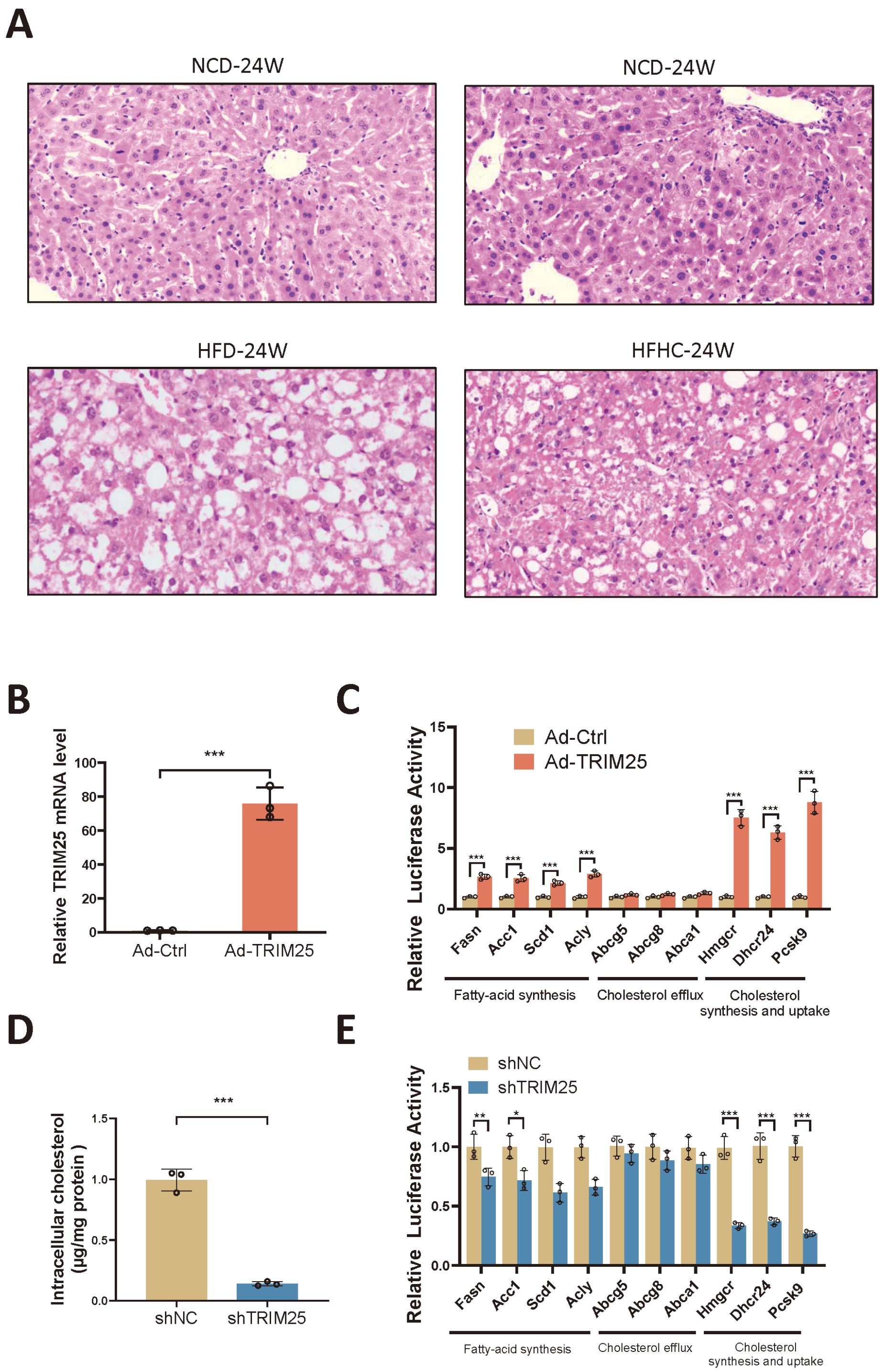

Figure S2. (A) Liver H&E staining images of MASH model mice induced by HFD and HFHC diets and normal chow diet mice. **(B)** After transfecting MIHA cells with Ad-TRIM25, the overexpression efficiency of TRIM25 was detected by RT-qPCR. **(C)** After overexpressing TRIM25 in MIHA cells, RT-qPCR was used to detect the expression levels of genes related to fatty acid synthesis, cholesterol efflux, and cholesterol synthesis. **(D)** After transfecting MIHA cells with shTRIM25, the knockdown efficiency of TRIM25 was detected by RT-qPCR. **(E)** After knocking down TRIM25 in MIHA cells, RT-qPCR was used to detect the expression levels of genes related to fatty acid synthesis, cholesterol efflux, and cholesterol synthesis. *$p < 0.05$, **$p < 0.01$, and ***$p < 0.001$.

Figure S3

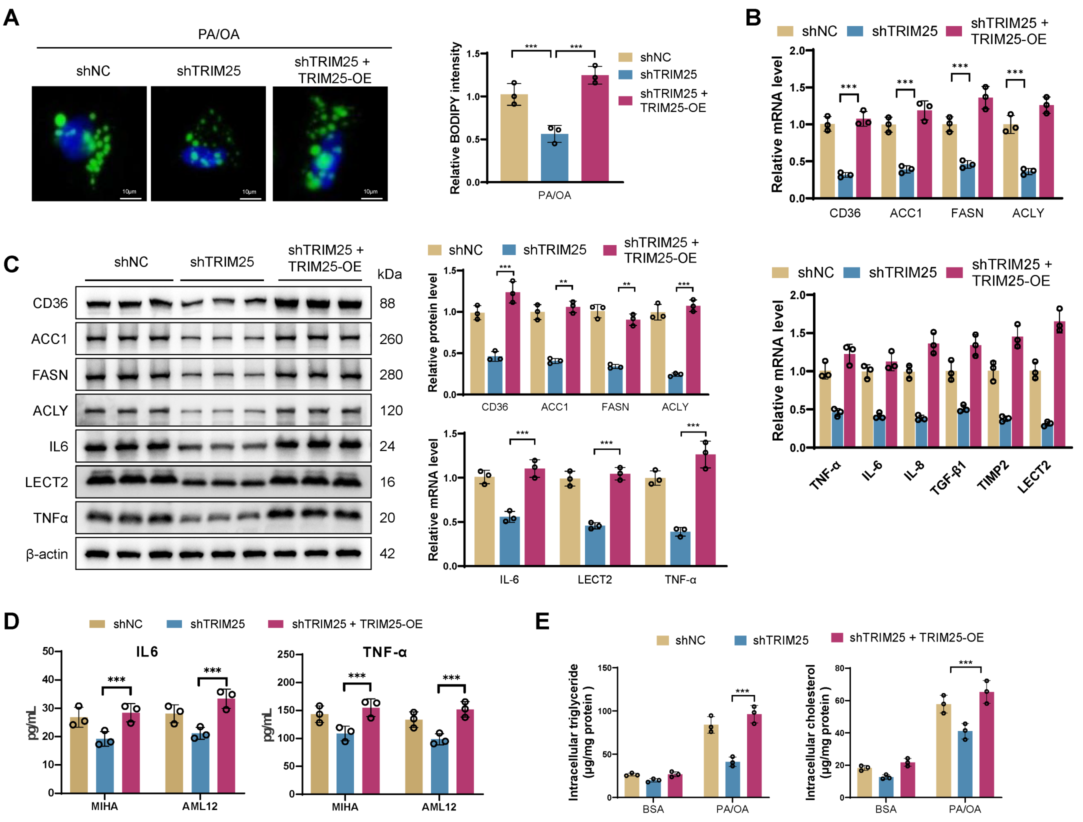

Figure S3. **(A)** Lipid droplet content was assessed using BODIPY staining in MIHA cells with TRIM25 knockdown and in TRIM25 knockdown cells with TRIM25 re-expression. Representative images and quantification are shown. **(B)** Relative mRNA expression levels of lipid metabolism-related genes in MIHA cells were determined by qRT-PCR. Data were normalized to GAPDH expression. **(C)** Protein expression levels of lipid metabolism-related proteins in MIHA cells were analyzed by western blotting. Representative blots and quantification are shown. **(D)** Levels of IL-6 and TNF-α in the cell culture medium were measured by ELISA in MIHA cells under the indicated conditions. **(E)** Intracellular TG and cholesterol contents in MIHA cells were quantified using specific assay kits. Data are presented as mean ± SD from at least three independent experiments. Statistical significance is indicated as *p < 0.05, **p < 0.01, and ***p < 0.001.

Figure S4

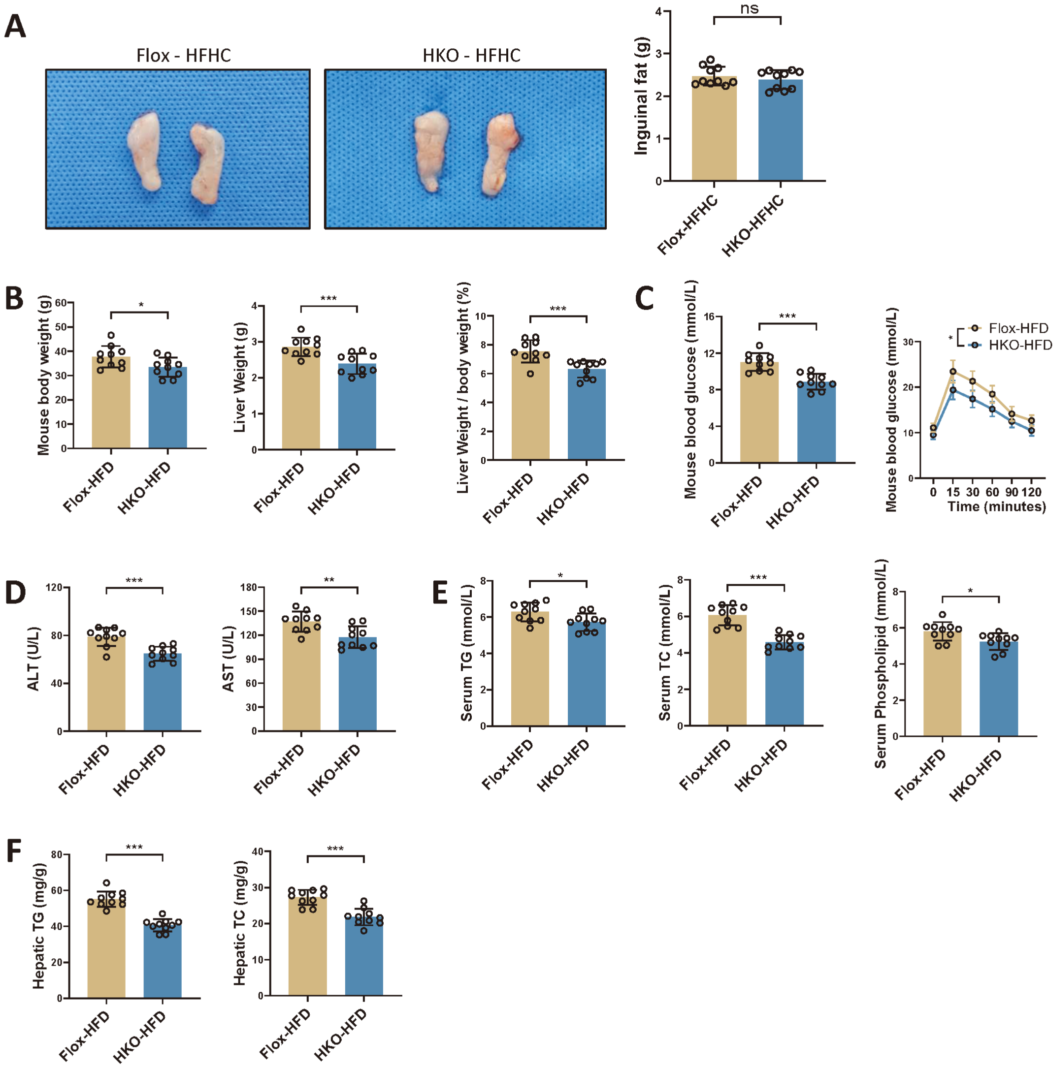

Figure S4. (A) Gross images and weight comparison of inguinal fat between Flox-HFHC and HKO-HFHC mice. **(B)** Body weight, liver weight, and liver weight/body weight ratio of Flox-HFHC and HKO-HFHC mice. **(C)** Blood glucose levels and oral glucose tolerance test of Flox-HFHC and HKO-HFHC mice. **(D)** Serum ALT and AST levels of Flox-HFHC and HKO-HFHC mice. **(E)** Serum triglyceride, total cholesterol, and phospholipid levels of Flox-HFHC and HKO-HFHC mice. **(F)** Triglyceride and total cholesterol levels in the liver tissues of Flox-HFHC and HKO-HFHC mice. *$p < 0.05$, **$p < 0.01$, and ***$p < 0.001$.

Figure S5

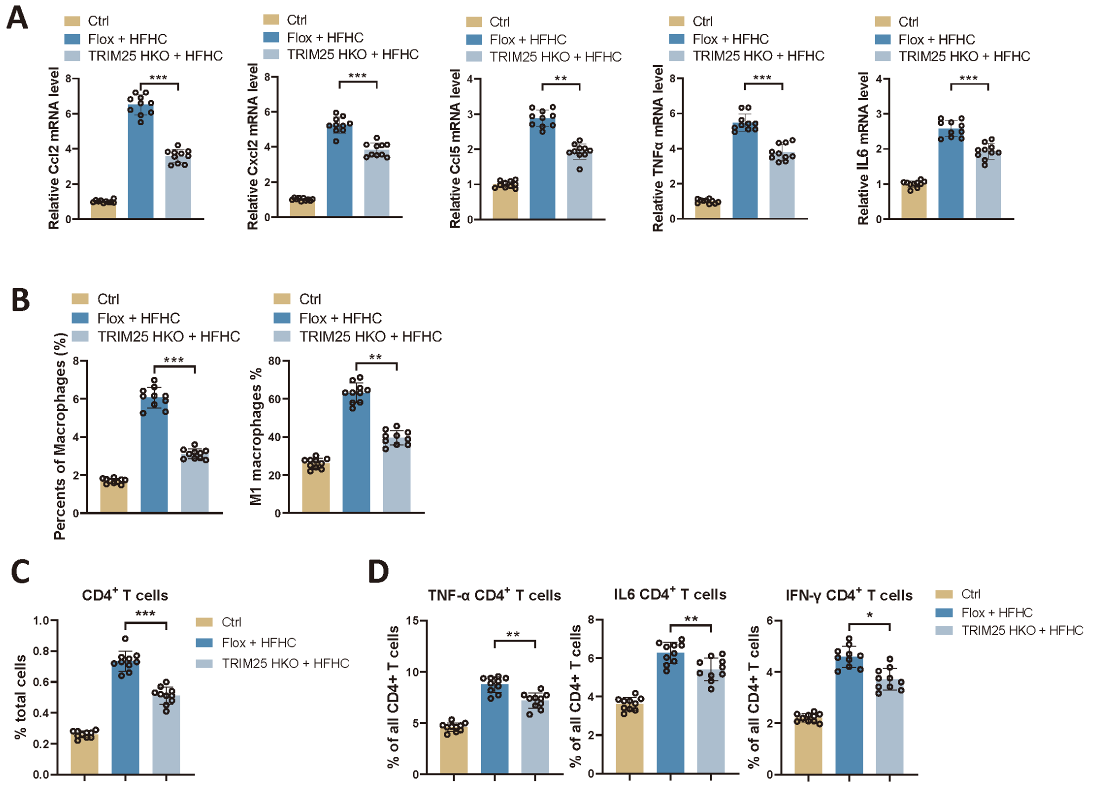

Figure S5. (A) The expression levels of fibrosis-related genes in the liver tissues of control, Flox-HFHC, and HKO-HFHC mice were detected by RT-qPCR. **(B)** The proportions of macrophages and the proportion of M1-type macrophages within the macrophage population in the liver tissues of control, Flox-HFHC, and HKO-HFHC mice were detected by flow cytometry. **(C)** The proportion of CD4+ T cells in the liver tissues of control, Flox-HFHC, and HKO-HFHC mice was detected by flow cytometry. **(D)** The proportions of TNF-α, IL-6, and IFN-γ positive cells within CD4+ T cells were detected by flow cytometry. *$p < 0.05$, **$p < 0.01$, and ***$p < 0.001$.

Figure S6

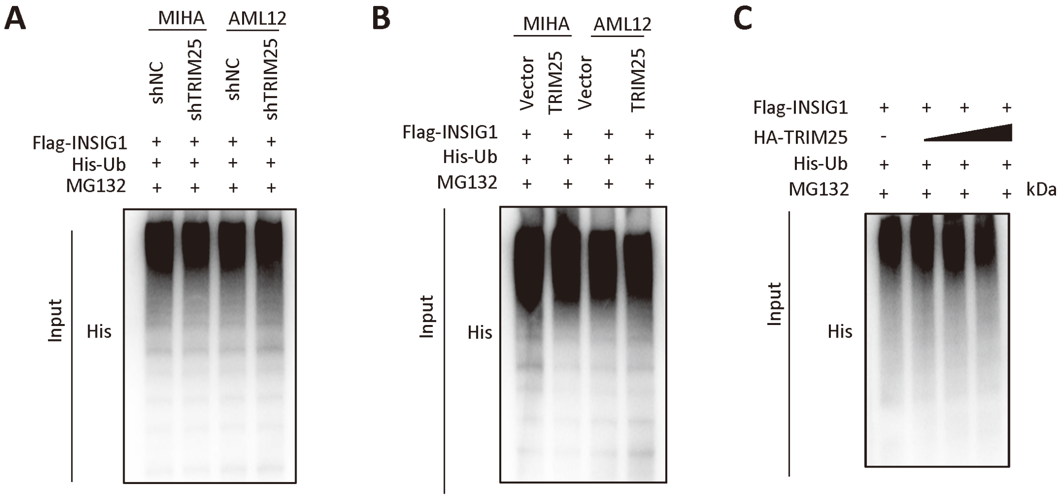

Figure S6. (A) The level of ubiquitination in the input group proteins was detected in the experiment of Figure 5F. **(B)** The level of ubiquitination in the input group proteins was detected in the experiment of Figure 5G. **(C)** The level of ubiquitination in the input group proteins was detected in the experiment of Figure 5I.

Figure S7

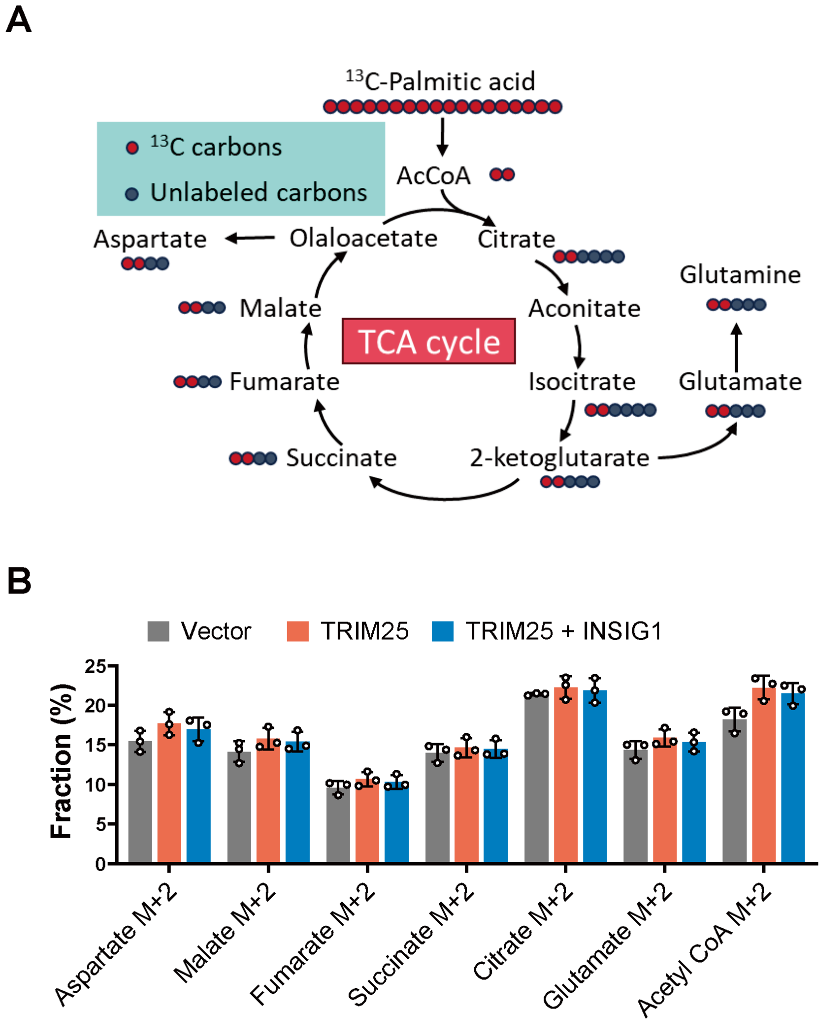

Figure S7. (A) schematic diagram showing the distribution of carbon atoms from ^{13}C-labeled palmitic acid participating in the TCA cycle. Control group, TRIM25 overexpression group, and TRIM25+INSIG1 overexpression MIHA cells were incubated with ^{13}C-labeled palmitic acid for 48 hours. **(B)** The distribution of ^{13}C-labeled products through the TCA cycle.

Figure S8

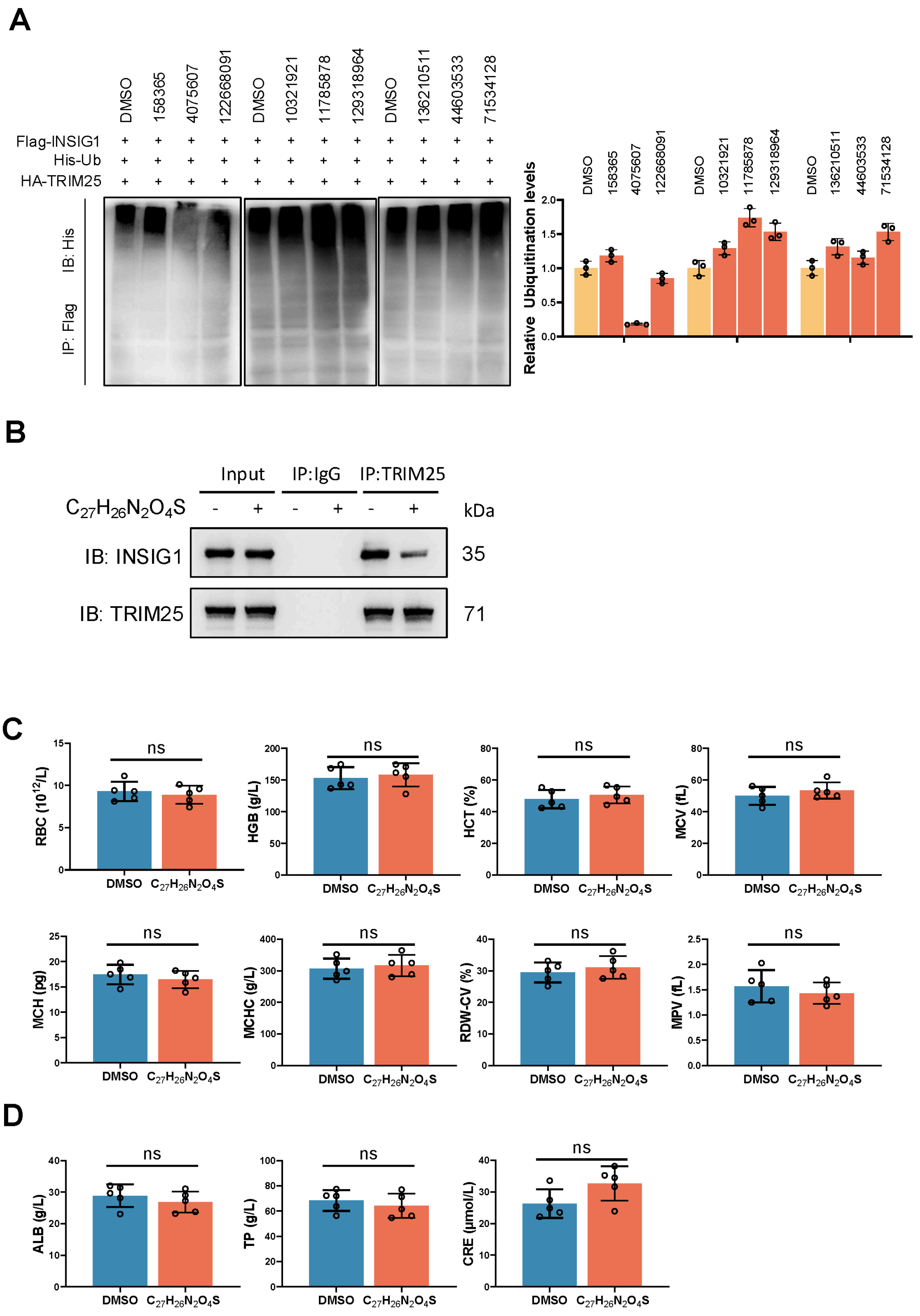

Figure S8. (A) Detect the ubiquitination level of INSIG1 after adding different small-molecule compounds. **(B)** IP was performed to examine the protein-protein interaction between TRIM25 and INSG1 in MIHA cells treated with or without $C_{27}H_{26}N_2O_4S$. TRIM25 was immunoprecipitated, and the levels of INSG1 in the TRIM25 pull-down complexes were analyzed by western blotting. **(C)** After administering an injection of $C_{27}H_{26}N_2O_4S$ at a dosage of 1 g/kg to C57BL/c mice, the relevant blood indicators of the mice were measured. **(D)** After injecting C57BL/c mice with $C_{27}H_{26}N_2O_4S$ at a dosage of 1 g/kg, test the relevant liver and kidney function indicators of the mice.

Figure S9

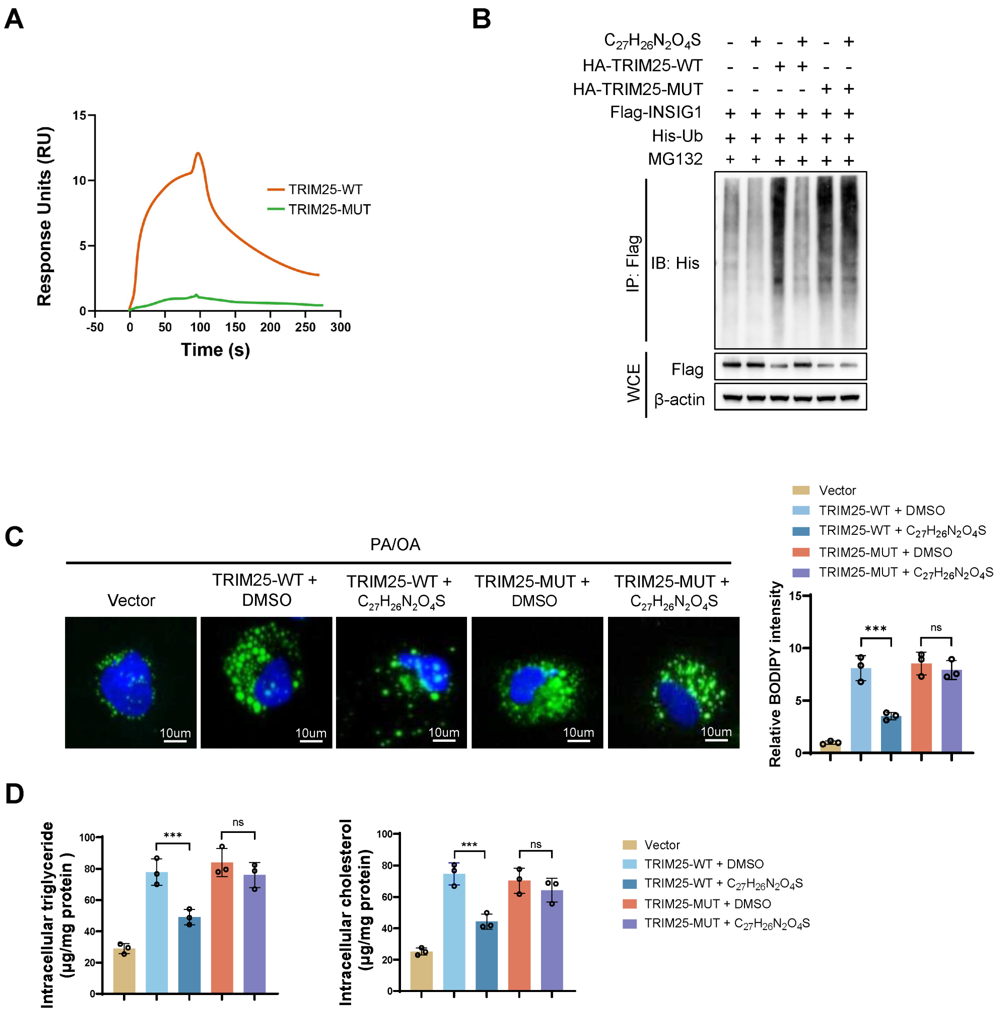

Figure S9. (A) SPR. **(B)** Assess the impact of TRIM25-WT or TRIM25-MUT on the ubiquitination levels of INSG1, experiments were conducted with and without the treatment of $C_{27}H_{26}N_2O_4S$. **(C)** The effects of TRIM25-WT or TRIM25-MUT on lipid accumulation in MIHA cells were evaluated in the presence or absence of $C_{27}H_{26}N_2O_4S$ treatment. **(D)** The influence of TRIM25-WT or TRIM25-MUT on triglyceride and cholesterol levels in MIHA cells was examined with or without C27H26N2O4S treatment. $*p < 0.05$, $**p < 0.01$, and $***p < 0.001$.

Figure S10

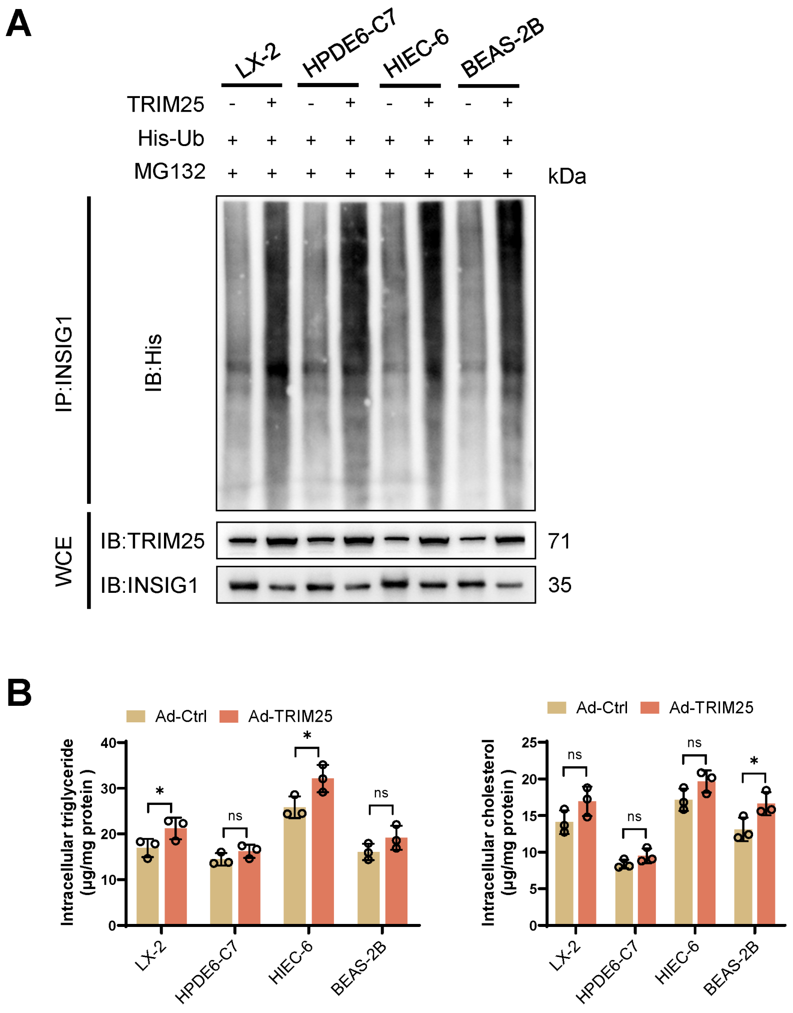

Figure S10. (A) Overexpression of TRIM25 in LX-2, HPDE6-C7, HIEC-6, and BEAS-2B cell lines also led to an increase in the ubiquitination levels of INSIG1. **(B)** The potential of TRIM25 to elevate intracellular triglyceride and cholesterol levels was assessed in LX-2, HPDE6-C7, HIEC-6, and BEAS-2B cell lines.

Figure S11

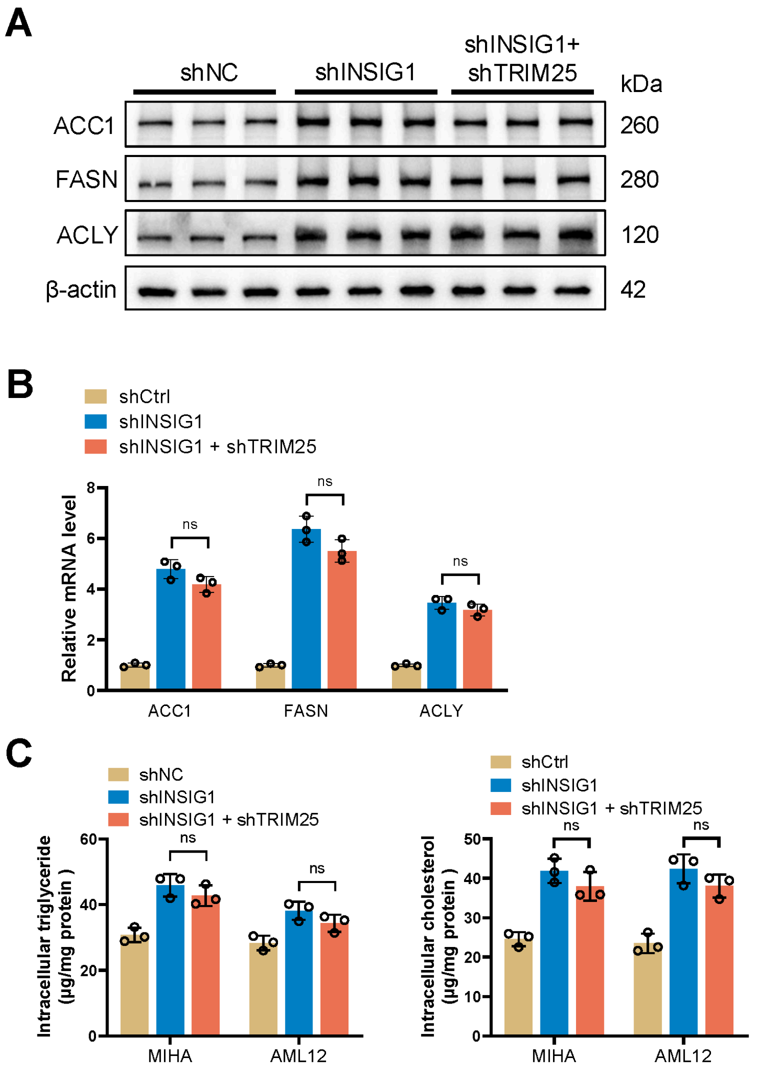

Figure S11. (A) Western blot analysis was performed to determine whether TRIM25 knockdown could still downregulate the protein levels of FASN, ACC1, and ACLY in INSIG1-depleted MIHA cells. **(B)** qRT-PCR was conducted to examine whether TRIM25 knockdown could still reduce the mRNA levels of FASN, ACC1, and ACLY in INSIG1-depleted MIHA cells. **(C)** The effect of TRIM25 on intracellular triglyceride and cholesterol levels was evaluated in INSIG1-depleted MIHA cells.

Figure S12

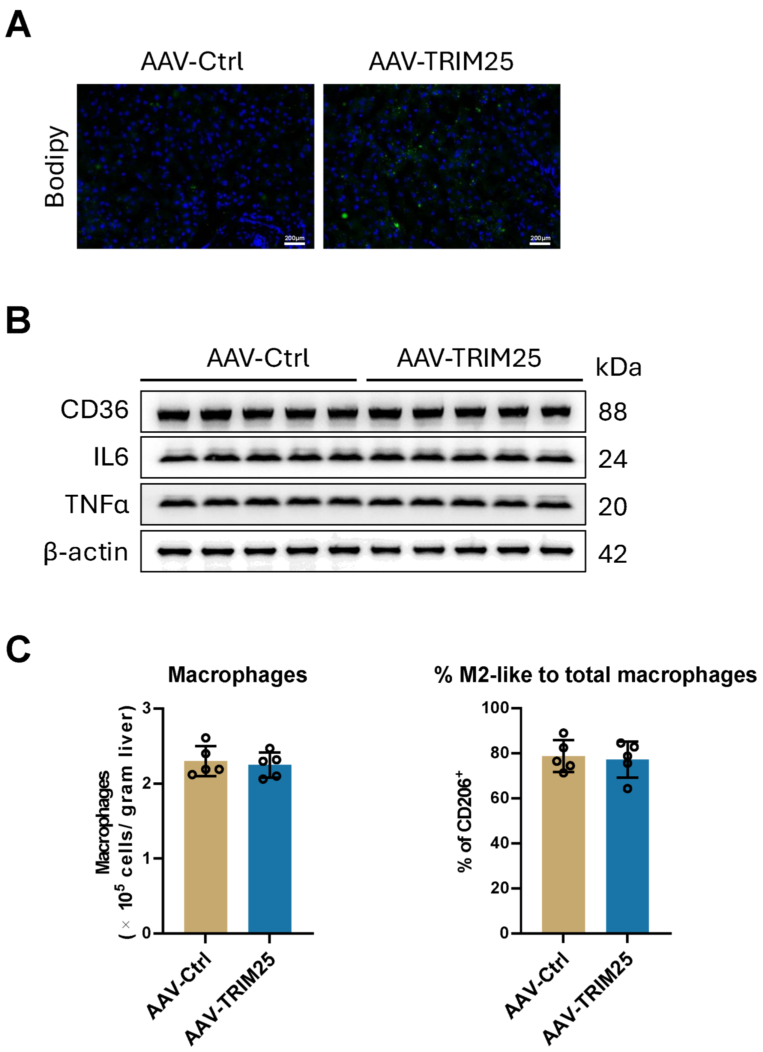

Figure S12. (A) BODIPY staining was performed to assess lipid droplet content in the livers of C57 mice after 4 weeks of HFHC diet induction. **(B)** The expression levels of related genes were confirmed to remain unchanged following hepatic overexpression of TRIM25 via AAV-TRIM25. **(C)** In the early MASH model, hepatic macrophage content and the proportion of M2-like macrophages remained unaltered following AAV-TRIM25 injection.

Table. S1 PCR primers.

Target genes	Primer types	Primer sequences
TRIM25-human	Forward Primer	AATCGGCTGCGGGAATTTTTC
	Reverse Primer	TCTCACATCATCCAGTGCTCT
TRIM25-mouse	Forward Primer	ATGGCTCAGGTAACAAGGGAG
	Reverse Primer	GGGAGCAACAGGGGTTTTCTT
actin-human	Forward Primer	CATGTACGTTGCTATCCAGGC
	Reverse Primer	CTCCTTAATGTCACGCACGAT
actin-mouse	Forward Primer	GTGACGTTGACATCCGTAAAGA
	Reverse Primer	GCCGGACTCATCGTACTCC
CD36-human	Forward Primer	GGCTGTGACCGGAACTGTG
	Reverse Primer	AGGTCTCCAACTGGCATTAGAA
ACC1-human	Forward Primer	ATGTCTGGCTTGCACCTAGTA
	Reverse Primer	CCCCAAAGCGAGTAACAAATTCT
ACC1-mouse	Forward Primer	CTCCCGATTCATAATTGGGTCTG
	Reverse Primer	TCGACCTTGTTTTACTAGGTGC
FASN-human	Forward Primer	AAGGACCTGTCTAGGTTTGATGC
	Reverse Primer	TGGCTTCATAGGTGACTTCCA
FASN-mouse	Forward Primer	GGAGGTGGTGATAGCCGGTAT
	Reverse Primer	TGGGTAATCCATAGAGCCCAG
TNFα-human	Forward Primer	CCTCTCTCTAATCAGCCCTCTG
	Reverse Primer	GAGGACCTGGGAGTAGATGAG
IL6-human	Forward Primer	ACTCACCTCTTCAGAACGAATTG
	Reverse Primer	CCATCTTTGGAAGGTTCAGGTTG
Interleukin 8	Forward Primer	TTTTGCCAAGGAGTGCTAAAGA
	Reverse Primer	AACCCTCTGCACCCAGTTTTC
TGFB1-human	Forward Primer	GGCCAGATCCTGTCCAAGC
	Reverse Primer	GTGGGTTTCCACCATTAGCAC
TIMP2-human	Forward Primer	AAGCGGTCAGTGAGAAGGAAG
	Reverse Primer	GGGGCCGTGTAGATAAACTCTAT
LECT2-human	Forward Primer	TGGGCCAGGAGAAACCTTATC
	Reverse Primer	CAAGGGCAATAGAGTTCCAAGTT
SCD1-mouse	Forward Primer	TTCTTGCGATACACTCTGGTGC
	Reverse Primer	CGGGATTGAATGTTCTTGTCGT
ACLY-mouse	Forward Primer	ACCCTTTCACTGGGGATCACA
	Reverse Primer	GACAGGGATCAGGATTTCCTTG
ABCG5-mouse	Forward Primer	AGAGGGCCTCACATCAACAGA
	Reverse Primer	CTGACGCTGTAGGACACATGC
ABCG8-mouse	Forward Primer	CTGTGGAATGGGACTGTACTTC
	Reverse Primer	GTTGGACTGACCACTGTAGGT
ABCA1-mouse	Forward Primer	GCTTGTTGGCCTCAGTTAAGG
	Reverse Primer	GTAGCTCAGGCGTACAGAGAT

Table. S2 Detailed information on the antibodies used in the experiments.

Antibody name	Cat No.	Product no.	Ratio of concentration
TRIM25 Polyclonal antibody	12573-1-AP	Proteintech	WB : 1:500-1:2000<br>IP : 2.0 ug for 2.0 mg of total protein lysate<br>IF : 1:100
Beta Actin Recombinant antibody	81115-1-RR	Proteintech	1:5000
CD36 Polyclonal antibody	18836-1-AP	Proteintech	WB : 1:1000
ACC1 Polyclonal antibody	21923-1-AP	Proteintech	WB : 1:2000
FASN Polyclonal antibody	10624-2-AP	Proteintech	WB : 1:10000
PPAR Gamma Polyclonal antibody	16643-1-AP	Proteintech	WB : 1:2000
IL-6 Polyclonal antibody	21865-1-AP	Proteintech	WB : 1:1000
Anti-LECT2	ab89835	abcam	WB : 1:1000
TNF Alpha Polyclonal antibody	26405-1-AP	Proteintech	WB : 1:1000
INSIG1 Polyclonal antibody	55282-1-AP	Proteintech	WB : 1:1000<br>IHC : 1:250<br>IF : 1:100
INSIG1 antibody	ABIN5703038	antibodies-online	IP:2.0 ug for 2.0 mg of total protein lysate
Flag Monoclonal antibody (Binds to FLAG® tag epitope)	66008-4-Ig	Proteintech	WB : 1:10000<br>IP : 2.0 ug for 2.0 mg of total protein lysate
HA tag Polyclonal antibody	51064-2-AP	Proteintech	WB : 1:5000-1:10000<br>IP : 2.0 ug for 2.0 mg of total protein lysate
GST Tag Monoclonal antibody	66001-2-Ig	Proteintech	WB : 1:10000<br>IP : 2.0 ug for 2.0 mg of total protein lysate
ACLY Monoclonal antibody	67166-1-Ig	Proteintech	WB : 1:1000
SREBF2 Polyclonal antibody	28212-1-AP	Proteintech	WB : 1:2000
SREBF2 Recombinant antibody	83403-1-RR	Proteintech	IF : 1:150
His-Tag Monoclonal antibody	66005-1-Ig	Proteintech	WB : 1:5000
Anti-SREBP2 antibody	ab30682	Abcam	WB : 1:1000

Table. S3 Results of Mass Spectrometry Analysis

Accession	Gene	Description	Mw(kDa)	Ctrl	TRIM25	log2(TRIM25/Ctrl)	Diff Sig
O15503	INSIG1	Insulin-induced gene 1 protein OS=Homo sapiens OX=9606 GN=INSIG1 PE=1 SV=2	29.987	25.3231	18.6852	-6.6379	--
P51668	UBE2D1	Ubiquitin-conjugating enzyme E2 D1 OS=Homo sapiens OX=9606 GN=UBE2D1 PE=1 SV=3	16.602	19.2973	25.2618	5.9645	++
P62837	UBE2D2	Ubiquitin-conjugating enzyme E2 D2 OS=Homo sapiens OX=9606 GN=UBE2D2 PE=1 SV=1	16.735	19.4344	25.2385	5.8041	++
P61077	UBE2D3	Ubiquitin-conjugating enzyme E2 D3 OS=Homo sapiens OX=9606 GN=UBE2D3 PE=2 SV=2	16.687	20.5562	26.1607	5.6045	++
P61088	UBE2N	Ubiquitin-conjugating enzyme E2 N OS=Homo sapiens OX=9606 GN=UBE2N PE=1 SV=3	17.138	17.8932	23.4517	5.5585	++
P17844	DDX5	Probable ATP-dependent RNA helicase DDX5 OS=Homo sapiens OX=9606 GN=DDX5 PE=1 SV=1	69.148	30.7036	25.3658	-5.3378	--
Q9UMR2	DD19B	ATP-dependent RNA helicase DDX19B OS=Homo sapiens OX=9606 GN=DD19B PE=1 SV=1	53.927	24.9737	19.8316	-5.1421	--
Q15910	EZH2	Histone-lysine N-methyltransferase EZH2 OS=Homo sapiens OX=9606 GN=EZH2 PE=1 SV=1	85.363	18.2281	23.3076	5.0795	++
O60260	PRKN	E3 ubiquitin-protein ligase parkin OS=Homo sapiens OX=9606 GN=PRKN PE=1 SV=1	51.641	19.4487	24.423	4.9743	++
P62979	RS27A	Ubiquitin-ribosomal protein eS31 fusion protein OS=Homo sapiens OX=9606 GN=RS27A PE=1 SV=1	17.965	18.8775	23.4925	4.615	+
Q08211	DHX9	ATP-dependent RNA helicase A OS=Homo sapiens OX=9606 GN=DHX9 PE=1 SV=3	140.958	19.6747	23.9709	4.2962	+
Q13263	TIF1B	Transcription intermediary factor 1-beta OS=Homo sapiens OX=9606 GN=TIF1B PE=1 SV=4	88.550	26.5655	22.4759	-4.0896	--
Q7Z2W4	ZCCHV	Zinc finger CCCH-type antiviral protein 1 OS=Homo sapiens OX=9606 GN=ZCCHV PE=1 SV=1	101.431	21.6957	25.672	3.9763	+
Q9BYM8	HOIL1	RanBP-type and C3HC4-type zinc finger-containing protein 1 OS=Homo sapiens OX=9606 GN=HOIL1 PE=1 SV=4	57.572	21.061	24.9541	3.8931	+
Q9UN86	G3BP2	Ras GTPase-activating protein-binding protein 2 OS=Homo sapiens OX=9606 GN=G3BP2 PE=1 SV=1	54.121	23.8325	20.0776	-3.7549	--
O00571	DDX3X	ATP-dependent RNA helicase DDX3X OS=Homo sapiens OX=9606 GN=DDX3X PE=1 SV=1	73.243	19.2065	22.9222	3.7157	+
Q9BZR9	TRIM8	3 ubiquitin-protein ligase TRIM8 OS=Homo sapiens OX=9606 GN=TRIM8 PE=1 SV=2	61.489	22.2705	25.6915	3.421	+
Q9Y2X8	UB2D4	Ubiquitin-conjugating enzyme E2 D4 OS=Homo sapiens OX=9606 GN=UB2D4 PE=3 SV=1	16.649	17.5407	20.9553	3.4146	+

P04637	P53	Cellular tumor antigen p53 OS=Homo sapiens OX=9606 GN=P53 PE=1 SV=3	43.653	18.9122	22.1506	3.2384	+
Q13283	G3BP1	Ras GTPase-activating protein-binding protein 1 OS=Homo sapiens OX=9606 GN=G3BP1 PE=1 SV=1	52.164	20.2875	23.3604	3.0729	+
P03372	ESR1	Estrogen receptor OS=Homo sapiens OX=9606 GN=ESR1 PE=1 SV=3	66.216	22.5411	25.5969	3.0558	+
P0CG48	UBC	Polyubiquitin-C OS=Homo sapiens OX=9606 GN=UBC PE=1 SV=1	77.039	19.117	22.0355	2.9185	+
P68036	UB2L3	Ubiquitin-conjugating enzyme E2 L3 OS=Homo sapiens OX=9606 GN=UB2L3 PE=1 SV=1	17.862	22.438	19.6675	-2.7705	-
Q13404	UB2V1	Ubiquitin-conjugating enzyme E2 variant 1 OS=Homo sapiens OX=9606 GN=UB2V1 PE=1 SV=2	16.495	20.7444	23.0836	2.3392	+
Q9UPY3	DICER	Endoribonuclease Dicer OS=Homo sapiens OX=9606 GN=DICER PE=1 SV=1	218.682	21.2977	18.9799	-2.3178	-
P60484	PTEN	Phosphatidylinositol 3,4,5-trisphosphate 3-phosphatase and dual-specificity protein phosphatase PTEN OS=Homo sapiens OX=9606 GN=PTEN PE=1 SV=3	47.166	18.8044	21.0876	2.2832	+
Q15170	TCAL1	Transcription elongation factor A protein-like 1 OS=Homo sapiens OX=9606 GN=TCAL1 PE=1 SV=1	18.641	21.4908	23.4736	1.9828	+
P67809	YBOX1	Y-box-binding protein 1 OS=Homo sapiens OX=9606 GN=YBOX1 PE=1 SV=1	35.924	21.7653	19.8027	-1.9626	-
Q96Q40	CDK15	Cyclin-dependent kinase 15 OS=Homo sapiens OX=9606 GN=CDK15 PE=1 SV=2	49.023	22.1769	20.2801	-1.8968	-
Q13887	KLF5	Krueppel-like factor 5 OS=Homo sapiens OX=9606 GN=KLF5 PE=1 SV=1	50.792	20.2075	21.92	1.7125	+
Q8N2K1	UB2J2	Ubiquitin-conjugating enzyme E2 J2 OS=Homo sapiens OX=9606 GN=UB2J2 PE=1 SV=1	28.898	20.2805	21.976	1.6955	+
Q99697	PITX2	Pituitary homeobox 2 OS=Homo sapiens OX=9606 GN=PITX2 PE=1 SV=1	35.370	23.4354	25.0657	1.6303	+
O14933	UB2L6	Ubiquitin/ISG15-conjugating enzyme E2 L6 OS=Homo sapiens OX=9606 GN=UB2L6 PE=1 SV=1	17.769	23.1931	21.767	-1.4261	-
P05161	ISG15	Ubiquitin-like protein ISG15 OS=Homo sapiens OX=9606 GN=ISG15 PE=1 SV=2	17.888	21.2439	22.612	1.3681	+
P46379	BAG6	Large proline-rich protein BAG6 OS=Homo sapiens OX=9606 GN=BAG6 PE=4 SV=1	119.409	26.0444	24.7071	-1.3373	-
P62256	UBE2H	Ubiquitin-conjugating enzyme E2 H OS=Homo sapiens OX=9606 GN=UBE2H PE=1 SV=1	20.655	22.9137	24.1788	1.2651	+

Q00839	HNRPU	Heterogeneous nuclear ribonucleoprotein U OS=Homo sapiens OX=9606 GN=HNRPU PE=1 SV=1	90.584	21.1046	22.3436	1.239	+
Q13206	DDX10	Probable ATP-dependent RNA helicase DDX10 OS=Homo sapiens OX=9606 GN=DDX10 PE=1 SV=1	100.888	21.5078	20.292	-1.2158	-
Q14839	CHD4	Chromodomain-helicase-DNA-binding protein 4 OS=Homo sapiens OX=9606 GN=CHD4 PE=1 SV=4	218.005	24.3823	25.4395	1.0572	+
Q1KMD3	HNRL2	Heterogeneous nuclear ribonucleoprotein U-like protein 2 OS=Homo sapiens OX=9606 GN=HNRL2 PE=1 SV=1	85.105	30.2666	29.2542	-1.0124	-
Q6ZTA4	TRI67	Tripartite motif-containing protein 67 OS=Homo sapiens OX=9606 GN=TRI67 PE=1 SV=1	83.823	26.4273	27.3939	0.9666	
Q8IUD6	RN135	E3 ubiquitin-protein ligase RNF135 OS=Homo sapiens OX=9606 GN=RN135 PE=1 SV=1	47.888	25.14	24.211	-0.929	
Q8IZD2	KMT2E	Inactive histone-lysine N-methyltransferase 2E OS=Homo sapiens OX=9606 GN=KMT2E PE=1 SV=3	204.965	28.8042	28.0015	-0.8027	
Q96C10	DHX58	ATP-dependent RNA helicase DHX58OS=Homo sapiens OX=9606 GN=DHX58 PE=1 SV=3	76.613	23.3968	24.1384	0.7416	
Q96GQ7	DDX27	Probable ATP-dependent RNA helicase DDX27 OS=Homo sapiens OX=9606 GN=DDX27 PE=1 SV=1	89.835	19.8985	20.6025	0.704	
Q9BUJ2	HNRL1	Heterogeneous nuclear ribonucleoprotein U-like protein 1 OS=Homo sapiens OX=9606 GN=HNRL1 PE=1 SV=1	95.739	25.5068	26.1526	0.6458	
Q9BUQ8	DDX23	Probable ATP-dependent RNA helicase DDX23 OS=Homo sapiens OX=9606 GN=DDX23 PE=1 SV=2	95.583	23.6948	23.0636	-0.6312	
Q9BYX4	IFIH1	Interferon-induced helicase C domain-containing protein 1 OS=Homo sapiens OX=9606 GN=IFIH1 PE=1 SV=1	116.689	22.4172	21.7933	-0.6239	
Q9BZY9	TRI31	E3 ubiquitin-protein ligase TRIM31 OS=Homo sapiens OX=9606 GN=TRI31 PE=1 SV=1	48.244	19.364	19.9188	0.5548	
Q9NY93	DDX56	Probable ATP-dependent RNA helicase DDX56 OS=Homo sapiens OX=9606 GN=DDX56 PE=1 SV=1	61.590	22.389	22.8908	0.5018	
Q9UHI6	DDX20	Probable ATP-dependent RNA helicase DDX20 OS=Homo sapiens OX=9606 GN=DDX20 PE=1 SV=2	92.241	22.0169	22.4817	0.4648	
Q9Y385	UB2J1	Ubiquitin-conjugating enzyme E2 J1 OS=Homo sapiens OX=9606 GN=UB2J1 PE=1 SV=2	35.199	21.0331	21.4637	0.4306	

Q969H0	FBXW7	F-box/WD repeat-containing protein 7 OS=Homo sapiens OX=9606 GN=FBXW7 PE=1 SV=2	79.663	22.8584	23.2521	0.3937
P61313	RL15	Large ribosomal subunit protein eL15 OS=Homo sapiens OX=9606 GN=RL15 PE=1 SV=2	24.146	26.8483	27.2237	0.3754
Q13330	MTA1	Metastasis-associated protein MTA1 OS=Homo sapiens OX=9606 GN=MTA1 PE=1 SV=2	80.786	23.3735	22.9996	-0.3739
Q8WVN8	UB2Q2	Ubiquitin-conjugating enzyme E2 Q2 OS=Homo sapiens OX=9606 GN=UB2Q2 PE=1 SV=2	42.818	21.3936	21.0449	-0.3487
O00425	IF2B3	Insulin-like growth factor 2 mRNA-binding protein 3 OS=Homo sapiens OX=9606 GN=IF2B3 PE=1 SV=2	63.705	23.7123	23.3838	-0.3285
O60885	BRD4	Bromodomain-containing protein 4 OS=Homo sapiens OX=9606 GN=BRD4 PE=1 SV=2	152.219	23.0533	23.3795	0.3262
O95793	STAU1	Double-stranded RNA-binding protein Staufen homolog 1 OS=Homo sapiens OX=9606 GN=STAU1 PE=1 SV=2	63.182	26.4946	26.1901	-0.3045
P13010	XRCC5	X-ray repair cross-complementing protein 5 OS=Homo sapiens OX=9606 GN=XRCC5 PE=1 SV=2	82.705	23.2489	23.5384	0.2895
P27694	RFA1	Replication protein A 70 kDa DNA-binding subunit OS=Homo sapiens OX=9606 GN=RFA1 PE=1 SV=2	68.138	28.696	28.4373	-0.2587
P49841	GSK3B	Glycogen synthase kinase-3 beta OS=Homo sapiens OX=9606 GN=GSK3B PE=1 SV=2	46.744	22.8807	23.1255	0.2448
P55072	TERA	Transitional endoplasmic reticulum ATPase OS=Homo sapiens OX=9606 GN=TERA PE=1 SV=2	89.322	21.0313	20.7987	-0.2326
Q05639	EF1A2	Elongation factor 1-alpha 2 OS=Homo sapiens OX=9606 GN=EF1A2 PE=1 SV=2	50.470	20.8545	20.6322	-0.2223
Q13501	SQSTM	Sequestosome-1 OS=Homo sapiens OX=9606 GN=SQSTM PE=1 SV=2	47.687	19.3495	19.1342	-0.2153
Q14498	RBM39	RNA-binding protein 39 OS=Homo sapiens OX=9606 GN=RBM39 PE=1 SV=2	59.380	24.928	24.7197	-0.2083
Q14694	UBP10	Ubiquitin carboxyl-terminal hydrolase 10 OS=Homo sapiens OX=9606 GN=UBP10 PE=1 SV=2	87.134	25.0329	24.8407	-0.1922
Q15029	U5S1	116 kDa U5 small nuclear ribonucleoprotein component OS=Homo sapiens OX=9606 GN=U5S1 PE=1 SV=2	109.436	24.3719	24.1949	-0.177
